# Structural Characteristics of *Rehmannia glutinosa* Polysaccharides Treated Using Different Decolorization Processes and Their Antioxidant Effects in Intestinal Epithelial Cells

**DOI:** 10.3390/foods11213449

**Published:** 2022-10-31

**Authors:** Heng Ren, Zhongyuan Li, Rui Gao, Tongxi Zhao, Dan Luo, Zihao Yu, Shuang Zhang, Chen Qi, Yaqi Wang, Hanzhen Qiao, Yaoming Cui, Liping Gan, Peng Wang, Jinrong Wang

**Affiliations:** College of Biology Engineering, Henan University of Technology, Zhengzhou 450001, China

**Keywords:** *Rehmannia glutinosa*, polysaccharides, structure, antioxidant activity

## Abstract

Polysaccharide decolorization is a key determinant of polysaccharide structure. In this study, two purified *Rehmannia glutinosa* polysaccharides, RGP−1−A and RGP−2−A, were obtained after decolorization using the AB-8 macroporous resin and H_2_O_2_, respectively. RGP−1−A (molecular weight (Mw) = 18,964 Da) and RGP−2−A (Mw = 3305 Da) were acidic and neutral heteropolysaccharides, respectively, and were both polycrystalline in structure. FTIR analysis revealed that RGP−1−A was a sulfate polysaccharide, while RGP−2−A had no sulfate group. Experiments on IPEC-1 cells showed that RGPs alleviated oxidative stress by regulating the Nrf2/Keap1 pathway. These findings were confirmed by the upregulation of Nrf2, NQO1, and HO-1; the subsequent increase in the levels of antioxidant indicators (SOD, LDH, CAT, and MDA); and the restoration of mitochondrial membrane potential. Overall, the antioxidant capacity of RGP−1−A was significantly higher than that of RGP−2−A. These results suggest that RGPs may be a potential natural antioxidant and could be developed into functional foods.

## 1. Introduction

Oxidative stress—a condition characterized by the excessive generation and accumulation of reactive oxygen species (ROS)—occurs owing to the increased production and poor clearance of ROS in living cells [1,2]. In the gastrointestinal tract, high levels of oxidative stress can cause a variety of gastrointestinal diseases. The gut, the largest organ of the immune system, is most vulnerable to oxidative attacks. Excessive ROS accumulation can impair signal transduction, causing oxidative damage to DNA, proteins, and lipids. These changes lead to molecular events such as autophagy, necrosis, and apoptosis [3,4], which can destroy the structure and function of the intestinal mucosa, increase the permeability of the intestinal barrier, and cause intestinal dysfunction. Such changes can induce the pathogenesis of colitis and even colon cancer [1].

*Rehmannia glutinosa* (RG) is a type of herb from the Scrophulariaceae family. In China, RG has a long history of medicinal and edible application. It has been cultivated on a large scale in the provinces of Henan, Shandong, Shanxi, and other places in China. Research has shown that RG can nourish Yin, enrich blood, and nourish essence [5]. More recently, RG has been used to develop functional foods and health supplements. Several trace elements and compounds—including polysaccharides (about 3.8%), catalpol (1–2%), glycosides (1–2%), and iridoid glycosides (0.2–0.5%)—have been isolated from RG [6,7]. Polysaccharides account for not only the largest proportion of saccharides in RG, but also for the largest proportions of all its chemical components. RG polysaccharides (RGPs) exert valuable biological activities; they regulate blood glucose and lipid levels [8], show anti-tumor activities [9], regulate immune function [10], and show anti-oxidant effects [5]. Hence, RGPs can provide benefits to human health and have a great potential for further development.

In recent years, polysaccharides have received wide attention owing to their unique physicochemical properties, functional characteristics, and biological activities, and are widely used in food, pharmaceutical, and cosmetic industries [11]. Numerous studies have shown that polysaccharides have excellent biological activities, such as anti-inflammatory [12], immunomodulatory [13], anti-tumor [14], antioxidant [15], and prebiotic activities [13]. However, natural plant materials such as roots, stems, leaves, and fruits contain a large amount of pigments. For example, the *Fritillaria unibracteata* polysaccharide from marine polysaccharides is brown [16], and the *Thesium chinense Turcz* polysaccharide from plant polysaccharides is dark brown [17]. Moreover, colored impurities may remain and contaminate fillers (Cellulose DE-52 and Sephadex Gel), increasing operating costs and interfering with structural and functional evaluation of polysaccharides [18]. Therefore, the pigment must be extracted or removed during the purification of polysaccharides. Several decolorization methods have been reported for isolating polysaccharides. These include activated carbon adsorption [17], macroporous resin adsorption [19], and H_2_O_2_ oxidation [18].

The biochemical mechanisms underlying each depigmentation process are different. The activated carbon adsorption method uses van der Waals forces to adsorb pigments onto the surface of activated carbon in order to achieve decolorization. This method is time-consuming, and the polysaccharide retention rate is low [20]. Macroporous resins can selectively enrich organic matter from a solution through physical adsorption because they are solid polymers with a macroporous structure and large specific surface area and are insoluble in water [21]. In aqueous solutions, H_2_O_2_ can ionize and release HO_2_^−^, which chemically attacks pigments. In an alkaline medium, the degree of ionization increases, and the decolorization effect is enhanced. Hence, a good decolorization effect is achieved [17]. However, one noteworthy issue is that HO_2_^−^ also attacks the glycosidic bonds of polysaccharides, which can lead to changes in their properties [22]. The extent to which the differences in decolorization methods for RGPs affect their structure and function is not clear.

In the present study, two decolorization methods—macroporous resin and H_2_O_2_ treatment—were used to obtain decolorized RGPs. The purpose of this study was to investigate the molecular properties, structural characteristics, and functional activities of RGPs that had been decolorized using H_2_O_2_ or the AB-8 macroporous resin. In this study, the molecular weight (Mw), monosaccharide composition, configuration, functional groups, and other structural characteristics of the RGP were analyzed, and the antioxidant capacity of the RGP was assessed to examine the association between the structure and the antioxidant activity.

## 2. Materials and Methods

### 2.1. Plant Products and Chemicals

The dried roots of RG were purchased from Wen County Agricultural Products Market, China. Monosaccharides were purchased from BBI Life Sciences, (Shanghai, China). Intestinal porcine epithelial cell line (IPEC-1) was purchased from Wuxi NEWGAINBIO Biological Company (Wuxi, China). All chemical reagents in the study were analytical reagents.

### 2.2. Preparation of RGP

The RGP extraction method was taken from previous studies and modified [23,24]. The rhizomes of RG were selected, cleaned, and sliced. The slices were dried at a constant temperature of 60 °C with an air blast. To obtain the crude RG powders, the slices were then crushed and passed through a 40-mesh sieve. The powdered sample (40 g) was mixed with 500 mL ultrapure water and treated with an ultrasonic device (200 W) at 65 °C for 1 h. The obtained solution was centrifuged 10 min (5000× *g*) and filtered. The supernatant was then concentrated, precipitated with absolute ethanol, and placed in the refrigerator at 4 °C for 12 h. The precipitate was then centrifuged and decolorized with the AB-8 macroporous resin (RGP-1) or H_2_O_2_ (RGP-2). The decolored polysaccharide solution was deproteinized using the Sevage method. Dialysis was performed in ultrapure water using a dialysis tube with an Mw cut-off value of 3500 Da. The sample solution was then freeze-dried to obtain the two RGP samples (RGP-1/RGP-2).

To further purify these polysaccharides, a modified version of a previously published protocol was used [25]. The crude polysaccharides (0.1 g) were dissolved in 10 mL ultrapure water and filtered via a 0.45 μm microporous membrane. They were then loaded onto a 2.6 × 40 cm DEAE-cellulose 52 column. Subsequently, NaCl solutions of different concentrations (0.1, 0.3, 0.5, 1 mol/L) were used to sequentially elute different fractions. The fractions eluted with ultrapure water were collected, concentrated at 65 °C, and lyophilized at 4 °C with a dialysis bag (3500 Da) to obtain the final polysaccharides (RGP−1−A/RGP−2−A). A Sephadex G-100 column (2.6 × 40 cm, Yuanye Bio-Technology, Shanghai, China) was used to further purify RGP−1−A and RGP−2−A. The polysaccharides were eluted with ultrapure water at 0.5 mL/min, and collected in different tubes, then the polysaccharide content in each collection tube was analyzed by the phenol sulfuric acid method, and a concentration curve was drawn. The tube corresponding to the elution peak was selected. Then, the solution was concentrated under low pressure and freeze-dried to obtain the pure polysaccharides RGP−1−A and RGP−2−A.

### 2.3. Molecular Properties of Polysaccharides

#### 2.3.1. Chemical Composition Analysis

The chemical compositions of RGP−1−A and RGP−2−A were analyzed. The phenol-sulfuric acid method was used to determine the total sugar content in the RGP [26], the carbazole method to determine its glyoxylate content [27], and the barium chloride/gelatinization method to determine the sulfate group content [26].

#### 2.3.2. Composition of Monosaccharides

A Shimadzu LC-20AD system (Shimadzu, Japan) was used to examine the monosaccharide content of RGP−1−A and RGP−2−A. An HP-5 capillary Xtimate C18 column (4.6 × 200 mm, 5 μm, Agilent Technology, California, USA) was used to separate and detect the monosaccharides, as described previously. The sample (5 mg) was added to a 5 mL ampoule, TFA (2.0 mL, 2 mol/L) was added to a 5 mL ampoule, the tube was sealed and acid digested at 110 °C for 4 h. We removed and evaporated the TFA and added 2.0 mL of water to re-dissolve. We precisely aspirated 0.25 mL of sample solution into a 5 mL EP tube, added NaOH (0.25 mL, 0.6 mol/L) and PMP-methanol (0.5 mL, 0.4 mol/L), and allowed it to react for 1 h at 70 °C. After cooling in cold water for 10 min, HCl was added to neutralize the mixture, and then 1 mL of chloroform was added and vortexed for 1 min. The mixture was centrifuged at 3000 r/min for 10 min, and the supernatant was carefully extracted three times. The supernatant was taken and filtered using a 0.22 μm membrane and analyzed by HPLC (Shimadzu, Tokyo, Japan).

#### 2.3.3. Mw Distribution

The average Mw distribution and polydispersity (Mw/Mn) of the two RGPs were determined according to methods proposed in previous studies [28,29]. The Mw of the RGPs was determined using a Shimadzu LC20 HPLC system equipped with a Shimadzu RID-20 differential refractive index detector (Shimadzu, Tokyo, Japan). The analysis conditions were as follows: the mobile phase was 0.1 mol/L NaNO_3_ and 0.06% NaN_3_; flow rate, 0.6 mL/min; the column temperature, 35 ± 0.1 °C; and injection volume, 20 μL.

#### 2.3.4. Zeta-Potential Analysis

Zeta-potentials were determined using a ZETASIZER Nano Series ZS 90 device (Malvern Instruments, Worcestershire, UK), as previously studied [30]. Briefly, samples were dissolved in deionized water at 1 mg/mL and ζ potentials were measured at 25 °C.

### 2.4. Structural Properties of Polysaccharides

#### 2.4.1. Fourier-Transform Infrared (FTIR) Spectroscopy Analysis

According to a previous study [31], this experiment was performed using an FTIR spectrometer (Nicolet 6700, Thermo Fisher, Waltham, MA, USA). In summary, 3 mg of RGP−1−A or RGP−2−A was mixed with KBr (100 mg) and then ground and pressed before analysis. The experimental wavelength range was 400~4000 cm^−1^.

#### 2.4.2. Scanning Electron Microscopy (SEM)

According to a previous study [32], the microstructure of the RGP−1−A and RGP−2−A samples was detected using SEM (jsm-6390/LV, NTC, Tokyo, Japan). Scanning was performed at an accelerating voltage of 15 kV at 3000× and 8000× magnification.

#### 2.4.3. X-ray Diffraction (XRD) Analysis

According to a previous study [33], the two polysaccharides were analyzed using an X-ray diffractometer (MiniFlex 600, Rigaku, Japan). The detection conditions were as follows: voltage, 40 kV; current, 100 mA; scanning range, 2θ 5°~80°; divergent slit and backscattering slit, 1.0 mm; receiving slit, 0.3 mm; step scanning; step size, 0.02°, scanning frequency, once per second.

#### 2.4.4. Nuclear Magnetic Resonance Spectroscopy (NMR) Analysis

First, 100 mg of RGP−1−A or RGP−2−A was dissolved in D_2_O and placed in the NMR tube. The ^1^H-NMR, ^13^C-NMR, and HSQC spectra were analyzed on a Bruker AVANCEIII HD 500 NMR spectrometer (Bruker, Rheinstetten, Germany).

#### 2.4.5. Thermogravimetric Analysis (TGA)

According to a previous study [34], the two polysaccharide samples (10 mg) were examined using a thermogravimetric analyzer (TA Q50, Perkin Elmer, Waltham, MA, USA). The samples, with an original temperature of 30 °C, were heated to 550 °C at a 10 °C/min rate, while the gas was switched to nitrogen and the flowing rate was 20.0 mL/min.

### 2.5. Antioxidant Capacity of RGP

#### 2.5.1. DPPH Radical Scavenging Activity (DRSA)

The DRSA of RGP−1−A and RGP−2−A was determined using a slight modification of a previous method [35]. Briefly, 2 mL of the polysaccharide sample solution with concentration gradient (0.5, 1, 2, 3, 4, and 5 mg/mL) was proposed with a 2 mL 0.1 mmol/L DPPH ethanol solution. The mixture was vortex mixed and then remained at 37 °C in the dark for 30 min, and the absorbance was determined at 517 nm. Ascorbic acid (Vc) was employed as positive control. The formula for clearing activity was as follows (Equation (1)):

(1) $$\mathrm{Scavenging}\;\mathrm{Activity}\;\left(\%\right)=\left(1-\frac{A_{1}-A_{2}}{A_{0}}\right)\times 100\%$$

A_0_: Absorbance of the control (2 mL anhydrous blank + 2 mL DPPH solution);

A_1_: Absorbance of the experimental group (2 mL polysaccharide solution/VC solution + 2 mL DPPH solution);

A_2_: Absorbance in the absence of DPPH (2 mL polysaccharide solution + 2 mL absolute ethanol).

#### 2.5.2. ABTS Radical Scavenging Activity (ARSA)

The ARSA of RGP−1−A and RGP−2−A was determined using a slight modification of a previously reported method [36]. Briefly, ABTS radicals were produced by mixing equal amounts of ABTS (7.4 mmol/L) and potassium persulfate (2.6 mmol/L). The tube containing the mixture was completely wrapped with tin foil and stored in the dark at room temperature for 12–14 h to generate large amounts of free radicals. The absorbance (734 nm) of the mixture was adjusted to 0.70 ± 0.02 with 95% ethanol. Then, 0.1 mL various concentrations (0.5, 1, 2, 3, 4, and 5 mg/mL) of polysaccharide samples were mixed with the ABTS solution (0.9 mL), and the absorbance at 734 nm was measured. Ascorbic acid was employed as the positive control. The clearing activity was calculated as follows (Equation (2)):

(2) $$\mathrm{Scavenging}\;\mathrm{Activity}\;\left(\%\right)=\left(1-\frac{A_{1}-A_{2}}{A_{0}}\right)\times 100\%$$

A_0_: Absorbance of the control group (0.1 mL ultrapure water + 0.9 mL ABTS solution);

A_1_: Absorbance of the experimental group (0.1 mL polysaccharide solution/VC solution + 0.9 mL ABTS solution);

A_2_: Absorbance in the absence of ABTS (0.1 mL polysaccharide solution/VC solution + 0.9 mL ultrapure water).

#### 2.5.3. Hydroxyl Radical (OH) Scavenging Activity (HRSA)

The HRSA of RGP−1−A and RGP−2−A was determined as described previously, with slight modifications [32,37]. First, 1 mL of the polysaccharide solution, 1 mL FeSO_4_ (6 mmol/L) and 1 mL salicylic acid-ethanol (9 mmol/L) were mixed, then, 1 mL H_2_O_2_ (9 mmol/L) was added to incubate for 30 min at 37 °C. The absorbance was measured at 510 nm. Ascorbic acid was employed as the positive control. The clearing activity was calculated as follows (Equation (3)):

(3) $$\mathrm{Scavenging}\;\mathrm{Activity}\;\left(\%\right)=\left(1-\frac{A_{1}-A_{2}}{A_{0}}\right)\times 100\%$$

A_0_: Absorbance of blank group (1 mL ultrapure water + 1 mL FeSO_4_ + 1 mL salicylic acid–ethanol solution + 1 mL H_2_O_2_);

A_1_: Absorbance of the experimental group (1 mL polysaccharide solution + 1 mL FeSO_4_ + 1 mL salicylic acid-ethanol solution + 1 mL H_2_O_2_);

A_2_: Absorbance in the absence of H_2_O_2_ (1 mL polysaccharide solution + 1 mL sterile water + 1 mL FeSO_4_ + 1 mL salicylic acid-ethanol solution).

### 2.6. Protection from H_2_O_2_-Induced Oxidative Damage in IPEC-1 Cells

#### 2.6.1. Cell Culture

The RPMI-1640 media with 10% fetal bovine serum and 1% penicillin/streptomycin (100×) was used to culture IPEC-1 cells at 37 °C under 5% CO_2_.

#### 2.6.2. Cell Viability Assay

Cell viability was assessed using the MTT test. The following procedures were used to treat IPEC-1 cells: (1) IPEC-1 cells were introduced into the 96-well plate at a density of 2 × 10^4^ cells per well and cultured overnight in an incubator. To induce an oxidative stress response, they were exposed to various doses of H_2_O_2_ (0, 0.1, 0.2, 0.3, 0.4, 0.5, 0.6, 0.7, 0.8, and 0.9 mmol/L) for 6 h. The optimal H_2_O_2_ concentration for developing the cellular model was selected. (2) IPEC-1 cells were treated with different concentrations of RGP−1−A and RGP−2−A (50, 100, 200, 400, and 800 μg/mL) for 12 h, and the effects of the polysaccharide concentrations on cell viability were detected. (3) H_2_O_2_ (0.6 mmol/L) was added to IPEC-1 cells pre-treated with RGP−1−A or RGP−2−A (100–400 μg/mL) for 12 h, and then incubated for an additional 6 h. Following the completion of the treatment periods, 20 μL MTT (5 mg/mL) was added to the 96-well plates. After 4 h of incubation, the medium was discarded, and each well was supplied with 150 μL of DMSO. The optical density was determined at 490 nm by a multipurpose microplate reader (Tecan, Switzerland) after the purple crystals were dissolved completely. The formula for cell viability was as shown in (Equation (4)):

(4) $$\mathrm{Cell}\;\mathrm{viability}\;\left(\%\right)=\frac{A_{1}}{A_{2}}\times 100\%$$

A_1_: Absorbance of the experimental group;

A_2_: Absorbance of the blank control group.

#### 2.6.3. Measurements of SOD, LDH, CAT, and MDA

To ensure complete adherence, IPEC-1 cells were added to a 6-well plate and cultured for 24 h. After being exposed to H_2_O_2_ (0.6 mmol/L) for 6 h, cells were treated with RGP−1−A and RGP−2−A solutions at three concentrations (100–400 μg/mL) for 12 h. Using an LDH test kit, the LDH concentrations in the cell supernatants were assessed. Cells were collected using a cell scraper and centrifuged (10,000× *g*, 5 min) at 4 °C. The collected IPEC-1 cells were lysed on ice and the supernatant was used to test the CAT, SOD, and MDA activity after centrifugation (10,000× *g*, 5 min, 4 °C).

#### 2.6.4. Mitochondrial Membrane Potential (MMP) Assay

The fluorescent probe JC-1 was used to examine the MMP in the IPEC-1 cells. In normal conditions, JC-1 forms an aggregate in the mitochondria and produces red fluorescence. When cells are damaged or apoptotic, JC-1 exists in the mitochondria in a monomeric state and shows green fluorescence. In 6-well plates, IPEC-1 cells (2 × 10^5^ cells/well) were treated with H_2_O_2_ (0.6 mM) for 6 h. Subsequently, the cells were exposed to 50–400 μg/mL of RGP−1−A/RGP−2−A for 12 h. The collected cells were washed carefully with PBS (pH = 7.4) and resuspended in JC-1 buffer solution before being incubated at 37 °C for 20 min. Mitochondrial JC-1 monomers (ex 514 nm and em 529 nm) and polymers (ex 585 nm and em 590 nm) were detected using an inverted fluorescence microscope (Echo Laboratories, RVL-100-G, San Diego, CA, USA) after three PBS washes.

##### 2.6.5. qRT-PCR Analysis

All the operations were conducted according to the manufacturer’s instructions; total RNA was extracted from IPEC-1 cells using the RNAiso plus reagent (Takara bio, Kusatsu, Japan). A reverse transcription kit (Takara bio, Japan) was used for cDNA synthesis based on the manufacturer’s instructions. The primer sequences are shown in Table S1. For real-time analysis, the SYBR qPCR master mix (Vazyme, Nanjing, China) and qTOWER 3G real-time fluorescence quantitative PCR system (Analytik Jena, Jena, Germany) were used. The PCR steps are shown in Table S2 below. Data were normalized based on GAPDH expression. The relative quantity of gene expression was calculated using the 2^-ΔΔCt^ method.

### 2.7. Statistical Analysis

The results are expressed as the mean ± standard deviation (SD). The differences between groups were compared using one-way analysis of variance (ANOVA), which was performed using the SPSS 20.0 program. Differences were deemed significant at *p <* 0.05.

## 3. Results and Discussion

### 3.1. Preparation of RGP

As can be seen in Figure S1, in the separation of RGPs using a DEAE-52 cellulose column, RGP-1 had three peaks for ultrapure water, 0.1 mol/L NaCl, and 0.3 mol/L NaCl, while RGP-2 had only two peaks for ultrapure water and 0.1 mol/L NaCl. The maximum eluted fractions (RGP−1−A and RGP−2−A) were then separately further purified and a single peak was obtained using size-exclusion chromatography on a Sephadex G-100 column. The high concentration of NaCl solution can elute down the higher negatively charged polysaccharide; however, some special groups in the polysaccharide, such as a sulfate group, carboxylic acid group, and so on, will cause it to be negatively charged [38]. This phenomenon may be caused by the attack of free radicals on the special groups in the polysaccharides during the H_2_O_2_ decolorization treatment, which in turn changes the group composition of the polysaccharides and thus causes their negative charge to decrease. Similar results were obtained in a previous study, where Cao et al. used UV/H_2_O_2_ to treat citrus pectin, which also led to a change in its negative charge [37].

### 3.2. MW

The results (Table 1) indicated that the molecular weights of RGP−1−A and RGP−2−A were 18,964 and 3305 Da, respectively, and the polydispersity indices (Mw/Mn) were 3.11382 and 1.33534, respectively, indicating that the decolorization effect of H_2_O_2_ caused the degradation of the RGPs, resulting in a much lower Mw and narrower polysaccharide distribution. The main reason for this phenomenon is that hydrogen peroxide generates free radicals, causing the breakage of glycosidic bonds in the main and/or side chains of polysaccharides. This view was also proven in previous studies, where Gong et al. found that the molecular weight of polysaccharides decreased with the increase in H_2_O_2_ treatment time by studying the effect of H_2_O_2_ degradation on polysaccharides at different times [22]. In addition, similar results were found in the study of Yao et al. Treatment of polysaccharides with H_2_O_2_ led to a decrease in the molecular weight of polysaccharides [39].

### 3.3. Monosaccharide Composition

**Figure 1:**
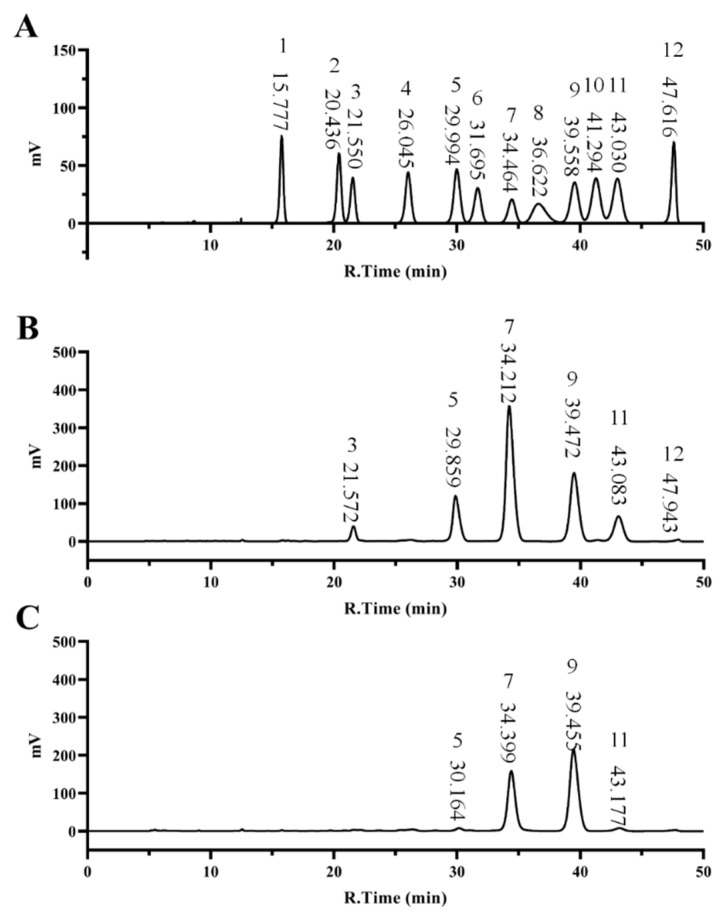
Monosaccharide composition of RGPs. Monosaccharide Standard Curve (**A**), RGP−1−A (**B**) and RGP−2−A (**C**). RGP−1−A: *Rehmannia glutinosa* polysaccharides decolorized by the AB-8 macroporous resin; RGP−2−A: *Rehmannia glutinosa* polysaccharides decolorized by H_2_O_2_.

As shown in Figure 1 and Table 2, RGP−1−A consisted mainly of glucose (45.335%), galactose (25.873%), arabinose (10.285%), and galacturonic acid (13.522%). In contrast, RGP−2−A was mainly composed of glucose (38.006%), galactose (56.461%), arabinose (2.47%), and galacturonic acid (1.196%). H_2_O_2_ decolorization did not change the monosaccharide composition of the two RGPs, but the content of the H_2_O_2_-decolorized galactose increased while that of the glucose and arabinose decreased, a phenomenon suggesting that the glycosidic bonds between glucose and arabinose molecules were preferentially cleaved by free radicals, similar to the results of Gong et al. [22]. The decrease in the content of galacturonic acid was also related to the attack of free radicals. In the study of Chen et al., the content of galacturonic acid in polysaccharides gradually decreased with the increase in H_2_O_2_ treatment time [40]. Therefore, after analysis of the above results we speculate that glucose, arabinose, and galacturonic acid were the sites of free radical attack.

### 3.4. Total Sugars, Glucuronic Acid and Sulfate Groups in RGP

As shown in Table 3, the total sugars, sulfate group, and glucuronic acid contents of RGP−1−A were 78.24 ± 0.52%, 11.83 ± 0.80%, and 19.02 ± 0.23%, respectively. In contrast, the total sugars, sulfate group, and glucuronic acid contents of RGP−2−A were 81.54 ± 0.79%, 2.79 ± 0.51%, and 1.1 ± 0.27%, respectively. The results for the glucuronic acid group were consistent with the monosaccharide composition, with RGP−1−A being higher than RGP−2−A, probably because of damage to the glucuronic acid group by free radicals released by H_2_O_2_. The results for the glucuronic acid group were consistent with the monosaccharide composition, with RGP−1−A being higher than RGP−2−A. There are two reasons for this; first, the destruction of the glucuronic acid group by free radicals released by H_2_O_2_, and then the dialysis of the decolorized polysaccharide, resulting in the escape of smaller molecules. Previous studies using H_2_O_2_-Vc-microwave degradation of *Grateloupia livida* sulfated polysaccharide and UV/H_2_O_2_ degradation of *Gracilaria lemaneiformis* sulfated polysaccharide both showed similar results in terms of decreased glucuronic acid content [22,41]. However, the variation in the sulfate group content differed considerably from the previous references. In the previous study, treatment of *Grateloupia livida* sulfate polysaccharides by the H_2_O_2_-Vc method did not reduce the content of the sulfate group [41]. In addition, treatment of *Sargassum fusiforme* polysaccharides by the ascorbic acid in combination with H_2_O_2_ increased the sulfate group [42]. In contrast, in this paper, there was a reduction in the sulfate group, probably owing to a reduction in the molecular weight of the polysaccharide after it was decolorized by H_2_O_2_ and some of the small molecules escaped during dialysis.

### 3.5. Zeta-Potential Analysis

The zeta-potential is defined as the potential of the hydrodynamic shear plane of a charged particle relative to the native solvent [43]. Because of the presence of carboxyl and sulfated groups in their structure, polysaccharides are negatively charged [44]. Among them, the zeta-potentials of RGP−1−A and RGP−2−A were −15.35 ± 0.45 and −8.76 ± 0.19 mV, respectively (Table 3). RGP−1−A with higher negative groups provided an inherently higher charge density and exhibited a stronger zeta-potential, in large part because of the anionic groups on the surface, which can lead to electrostatic repulsion and a rigid conformation. RGP−1−A with higher negative groups (sulfate groups, glucuronic acid groups) provided an inherently higher charge density and exhibited a stronger zeta-potential. In addition, the larger molecular weights of RGP−1−A may have longer glycoconjugates, and the glycoconjugates are less prone to collapse and are able to exhibit greater ionic strength, a phenomenon that has been mentioned in previous studies [45].

### 3.6. XRD Analysis

XRD is extremely helpful to determine the crystal structure of polysaccharides, which can yield valuable information on their expansibility, flexibility, tensile strength, and solubility [36]. Figure S2 shows the XRD patterns of the two types of polysaccharides. The peaks for RGP−1−A and RGP−2−A were similar between 5° and 90°. However, RGP−1−A and RGP−2−A had distinct broad peaks at 21°. The weak peaks indicated that these polysaccharides had very low crystallinity and an amorphous structure. However, the diffraction peak of RGP−1−A was wider and higher than that of RGP−2−A. We speculate that the free radicals generated by H_2_O_2_ attacked the glycosidic bonds, causing the polysaccharides to break from long chains into fragments, which, in turn, formed hydrogen bonds between the hydrophilic sites and increased crystallinity. This result was similar to the previous findings of Hu et al., who modified citrus pectin by degrading it using an ultrasonic NaHCO_3_-H_2_O_2_ system, and the crystallinity of the modified pectin increased [46].

### 3.7. FTIR Spectroscopy Analysis

**Figure 2:**
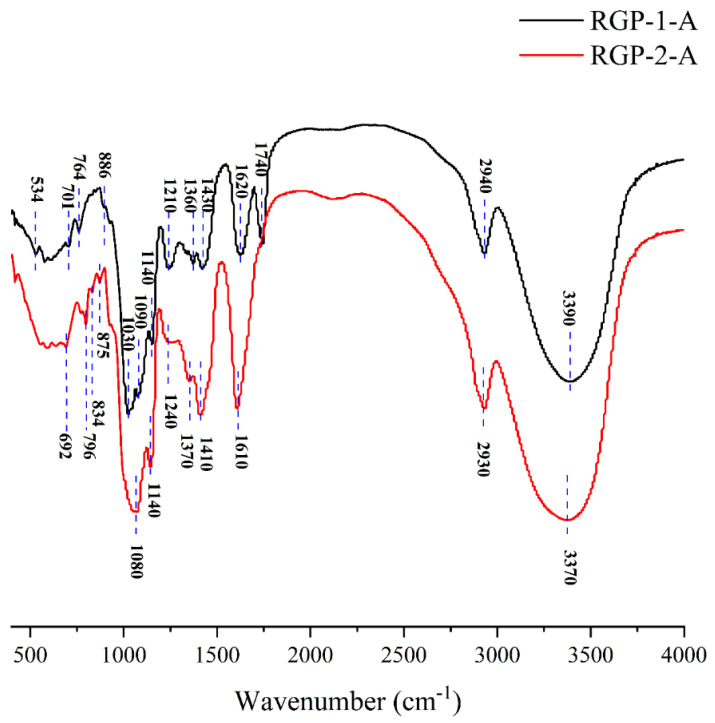
FTIR patterns of RGP−1−A and RGP−2−A. RGP−1−A: *Rehmannia glutinosa* polysaccharides decolorized by the AB-8 macroporous resin; RGP−2−A: *Rehmannia glutinosa* polysaccharides decolorized by H_2_O_2_.

FTIR is necessary for detecting the functional group composition of polysaccharides. In this study, FTIR was used to analyze the RGPs after decolorization using macroporous resin and H_2_O_2_. Figure 2 and Table 4 show the peaks and functional groups of the FTIR spectra of the two polysaccharides. The broad and strong absorption peaks near 3370 and 3390 cm^−1^ indicated the presence of free hydroxyl groups on RGP−1−A and RGP−2−A [18]. The peak at 2930 and 2940 cm^−1^ represented the tensile vibration of C−H bonds in the polysaccharide sugar ring [36]. The typical absorption peak at 1000–1100 cm^−1^ was related to the pyranose rings. In addition, absorption peaks were detected at 1140 cm^−1^ (RGP−1−A and RGP−2−A), which are associated with asymmetric C−O−S stretching vibrations [22,47]. Both RGP−1−A (1620, 1430 cm^−1^) and RGP−2−A (1610, 1410 cm^−1^) had resonance of the COO− group in their FTIR spectra. The 1740 cm^−1^ of RGP−1−A further indicated that there was glucuronic acid in RGP−1−A [48,49,50]. The sulfate group was present within both RGP−1−A (1360, 1140, 886 cm^−1^) and RGP−2−A (1370, 1140, 875, 834 cm^−1^) [22,47,51,52]. This apparent difference between functional groups in RGP−1−A and RGP−2−A may be due to the ability of H_2_O_2_ to degrade galacturonic acid, consistent with the findings on monosaccharide composition. These results suggested that H_2_O_2_ decolorization can destroy some glycosidic bonds in polysaccharides and create structural differences, as reported previously [22].

### 3.8. TGA

TGA can provide valuable information on the thermal weightlessness of materials, which is closely related to their molecular structure and reflects their thermostability. To some extent, thermal properties determine the applicability of polysaccharides [55]. As shown in the TGA images in Figure S3A,B, the loss rates of RGP−1−A and RGP−2−A were 6.888% and 4.377%, respectively, from room temperature to 125–150 °C. This was due to the volatilization of a small amount of solvent and the loss of bound water present in the pores of the polysaccharide [25]. Between 125 and 350 °C, the loss rates of RGP−1−A and RGP−2−A were 68.11% and 55.62%, respectively, which may be partly caused by the loss of sulfate groups (250–300 °C), amide groups (300–330 °C) and backbone decomposition [56]. Above 350 °C, the weight loss curve leveled off and the mass of polysaccharides decreased slowly because most of them had turned into carbonized structures after high temperature heating. The residues of RGP−1−A and RGP−2−A were 8.79% and 29.77%, respectively, indicating that RGP−2−A had good thermal stability. In previous studies, it was also found that polysaccharides with a higher content of sulfate groups were relatively weak in their thermal stability [55].

### 3.9. SEM

**Figure 3:**
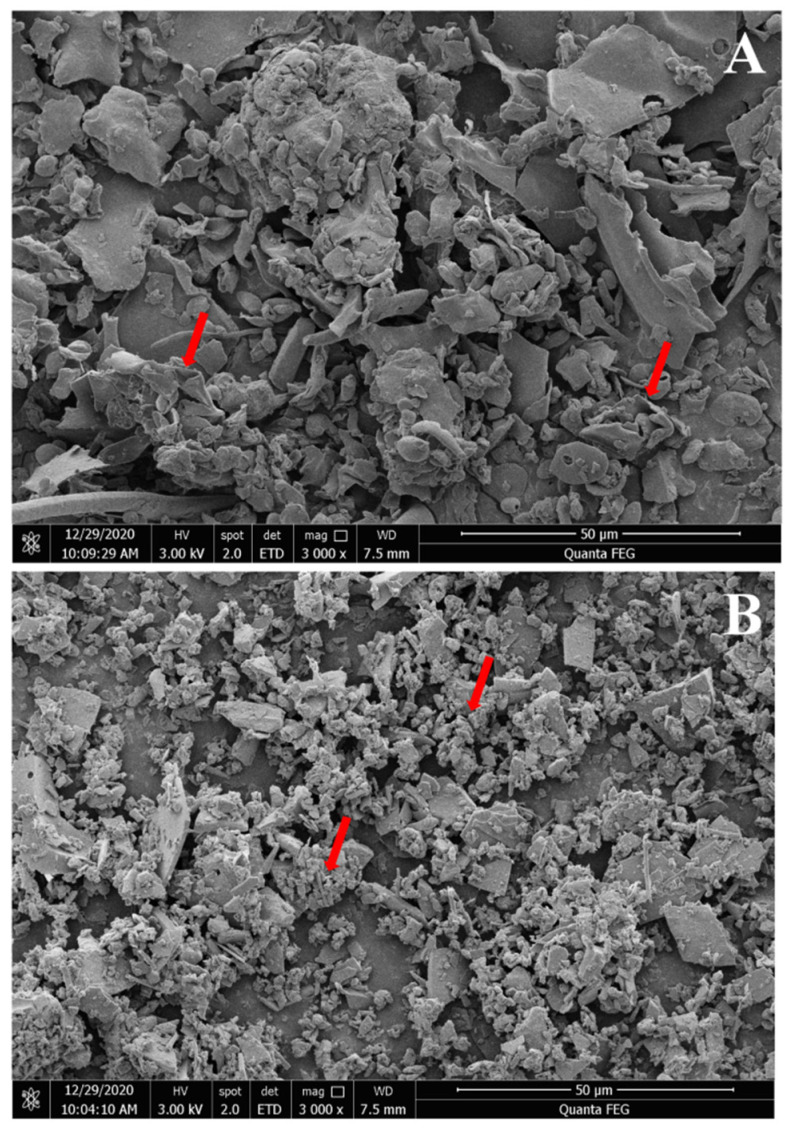
Scanning electron micrographs of two *Rehmannia glutinosa* polysaccharides. Magnifications of 3000× for RGP−1−A (**A**) and RGP−2−A (**B**). RGP−1−A: *Rehmannia glutinosa* polysaccharides decolorized by the AB-8 macroporous resin; RGP−2−A: *Rehmannia glutinosa* polysaccharides decolorized by H_2_O_2_. The red arrows indicate significant differences.

Three-dimensional images of the polysaccharides were obtained using SEM and were used to examine the surface morphology of the RGPs. As shown in the SEM images (Figure 3), RGP−1−A and RGP−2−A had different morphological features. The shape of RGP−1−A was mainly in the form of large irregular curly flakes, while the RGP−2−A after H_2_O_2_ decolorization was mainly in the form of small fine flakes. Combining the results of monosaccharide composition and molecular weight, we speculate that this change may be due to the difference in the SEM results of the two polysaccharides caused by the free radical attack on the polysaccharide glycosidic bond, and the breakage of the glycosidic bond induced a morphological change. Similar results were found in a previous study, where Chen et al. analyzed UV/H_2_O_2_-treated *Sargassum fusiforme* polysaccharides and found that UV/H_2_O_2_ treatment resulted in smaller and more uniform polysaccharide sizes [40].

### 3.10. NMR Analysis

**Figure 4:**
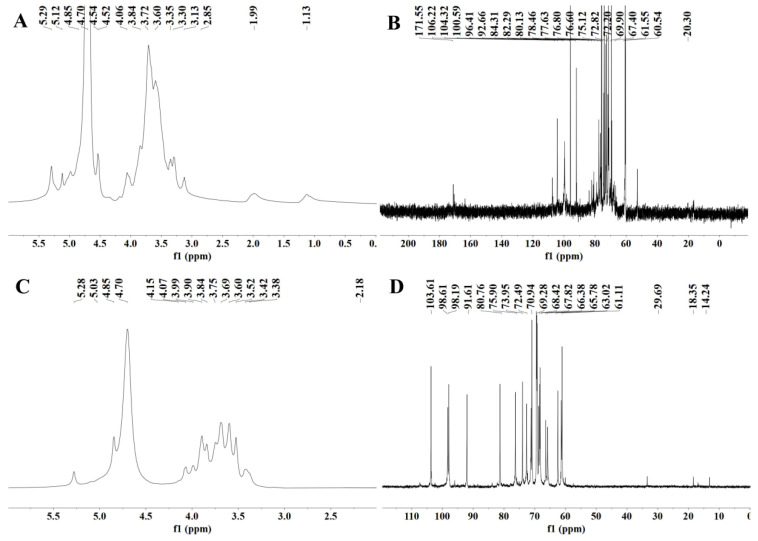
NMR spectra of two *Rehmannia glutinosa* polysaccharides. (**A**) ^1^H spectrum of RGP−1−A; (**B**) ^13^C spectrum of RGP−1−A; (**C**) ^1^H spectrum of RGP−2−A; (**D**) ^13^C spectrum of RGP−2−A. RGP−1−A: *Rehmannia glutinosa* polysaccharide decolorized by AB-8 macroporous resin, RGP−2−A: I polysaccharide decolorized by H_2_O_2_.

**Figure 5:**
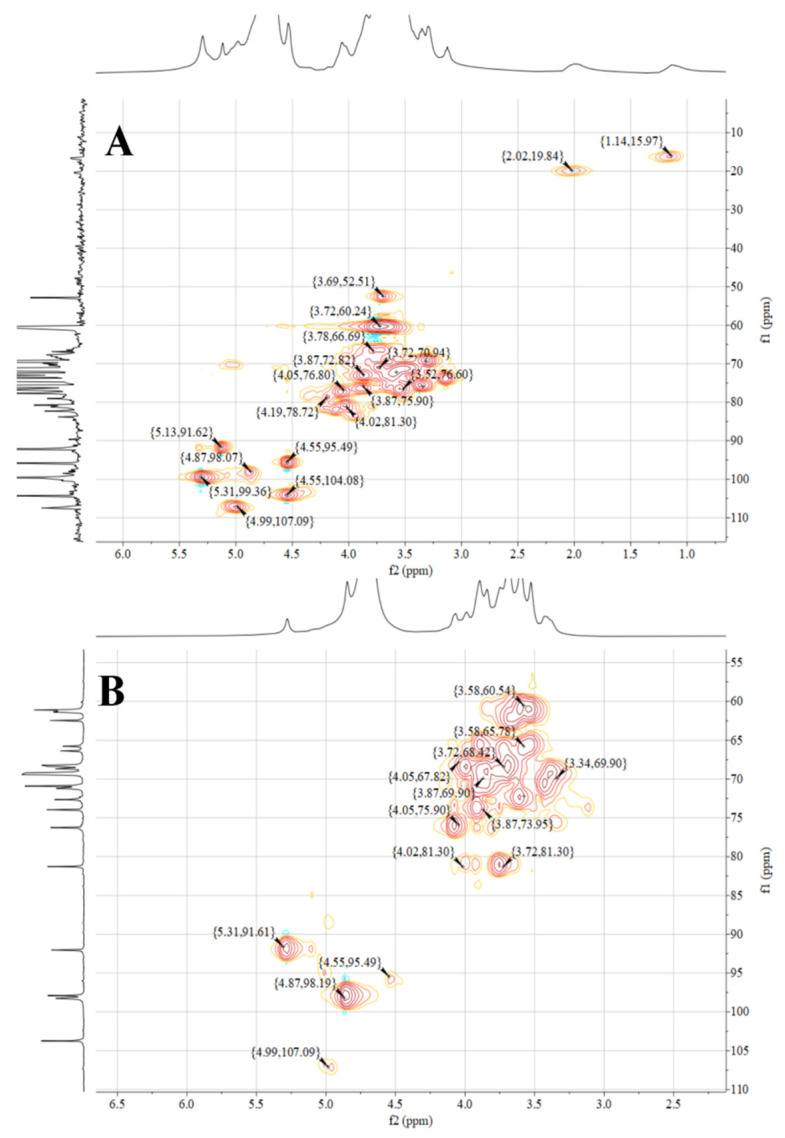
NMR spectra of two *Rehmannia glutinosa* polysaccharides. (**A**) HSQC spectrum of RGP−1−A; (**B**) HSQC spectrum of RGP−1−A. RGP−1−A: *Rehmannia glutinosa* polysaccharide decolorized by AB-8 macroporous resin, RGP−2−A: I polysaccharide decolorized by H_2_O_2_.

NMR is an indispensable tool in the structural analysis of polysaccharides. As seen in Figure 4A,B, the anomeric region was nearly in the range of 3.0 to 5.5 ppm, which is a typical signal for polysaccharides. The peak at 4.70 ppm was attributed to the chemical shift of D_2_O. Usually, the chemical shifts of hydrogen protons on α-glycoside residues and β-glycoside residues appear between 5.0 and 5.5 ppm and 4.0 and 5.0 ppm, respectively [57,58]. However, the ^1^H spectra 4.85−5.4 ppm and 4.4−4.6 ppm peaks were heavily stacked (Figure 4A,B), so that the specific chemical shifts could not be distinguished [59]. This phenomenon may be due to the low solubility of RGPs in D_2_O, resulting in poor dispersion of the NMR peaks and high noise values. The signal at 1.14 ppm was probably from rhamnose H-6 (Figure 4A). The methyl group was attributed to rhamnose C-6 [60], which was confirmed by the 1.14/15.97 ppm cross-peak in the HSQC spectrum (Figure 5A). The signal at 2.17 ppm was designated as a proton on the O-acetyl group (Figure 4A), and 20.26 ppm of the ^13^C signal was designated as a methyl carbon signal on the O-acetyl group (Figure 4B) [61]. In addition, an absorption peak was observed at 171.55 ppm, indicating the presence of glucuronic acid in the RGP, and the low-field signal was labeled as a carbonyl group on the O-acetyl group, i.e., C-6 of the glucuronic acid [62], which was consistent with the above results.

Figure 5 shows the NMR spectra of the HSQC of RGP−1−A and RGP−2−A. The signals at 4.99/107.09 ppm (RGP−1−A) and 4.99/107.09 ppm (RGP−2−A) were designated as α-L-Ara based on relevant studies and literature [63]. The signal at 4.55/104.08 ppm (RGP−1−A) was designated as β-D-Gal [64]. The signal at 4.87/99.36 ppm indicated the presence of α-Gal A [61]. The signals at 4.87/98.07 ppm (RGP−1−A) and 4.87/98.19 ppm (RGP−2−A) indicated the presence of α-D-Glc [60]. The signals at 4.55/95.49 ppm (RGP−1−A) and 4.55/95.49 ppm (RGP−2−A) indicated the presence of β-D-Glc [65]. The signals at 5.13/91.62 ppm (RGP−1−A) and 5.31/91.61 ppm (RGP−2−A) indicated the presence of α-D-Glc [64].

### 3.11. Antioxidant Capacity of RGP

**Figure 6:**
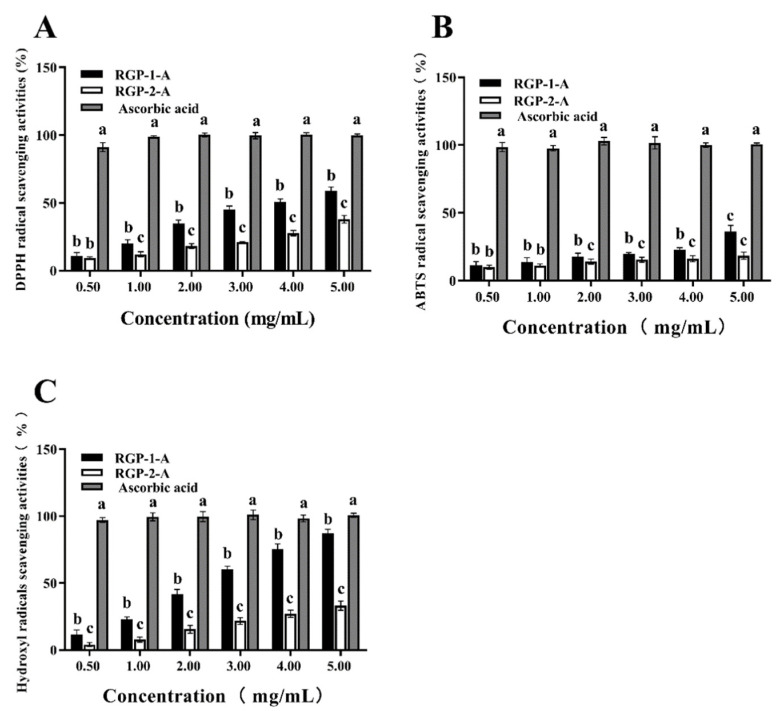
Anti-oxidative activity of *Rehmannia glutinosa* polysaccharides at different concentrations. The scavenging activity against (**A**) DPPH, (**B**) ABTS, and (**C**) OH was examined. RGP−1−A: *Rehmannia glutinosa* polysaccharides decolorized by the AB-8 macroporous resin; RGP−2−A: *Rehmannia glutinosa* polysaccharides decolorized by H_2_O_2_. Different lowercase letters on the bar graph indicate significant differences (*p <* 0.05).

DPPH radicals are relatively stable radicals containing one electron. Once these radicals react with polysaccharides/oxidants, the color of the DPPH solution changes [58]. The two RGPs showed obvious DRSA (Figure 6A). The DRSA of the RGPs within the experimental concentration range gradually improved with the increase in polysaccharide contents, exhibiting a dose-dependent effect. The scavenging rates of RGP−1−A and RGP−2−A against DPPH free radicals were 58.8 ± 2.9% and 36.9 ± 1.5%, respectively, when the polysaccharide concentrations were 5 mg/mL. Obviously, RGP−1−A showed greater DPPH scavenging activity than RGP−2−A (*p <* 0.01).

ABTS solutions are blue-green in color and become colorless when they react with antioxidants [66]. Within the range of experimental concentrations, as the concentration of polysaccharides increased, their scavenging ability against ABTS radicals also gradually increased in a dose-dependent manner (Figure 6B). When the polysaccharide concentration increased to 5 mg/mL, the ARSA values of RGP−1−A and RGP−2−A were 35.74 ± 3.98% and 16.725 ± 0.28%, respectively. Hence, RGP−1−A showed greater ARSA in the tested concentration range (*p <* 0.01).

OH· can cause severe oxidative damage to biological macromolecules, resulting in cell and tissue damage and ultimately organ dysfunction and aging [32]. As shown in Figure 6C, with the increase in polysaccharide concentration, the HRSA of the RGPs showed a significant upward trend (*p <* 0.01). A total of 5.0 mg/mL of RGP−1−A and RGP−2−A showed scavenging rates of 87.18 ± 1.24% and 33.11 ± 1.92%, respectively.

It is well-known that properties such as polysaccharide monosaccharide composition, molecular weight, structure, sulfate content, and glucuronic acid content have a great influence on the biological activity of polysaccharides [67,68,69]. The larger the molecular weight, the stronger the antioxidant capacity of the polysaccharide. In this paper, the molecular weight of RGP−1−A was much higher than that of RGP−2−A, and the antioxidant capacity of RGP−1−A was significantly higher than that of RGP−2−A. This was similar to the previous study by Ge et al. [68] in which the antioxidant capacity of *Herba Lophatheri* polysaccharides of different molecular weights was analyzed, and the higher the molecular weight of the polysaccharide, the higher the antioxidant capacity. Moreover, similar results were found in the study of Li et al. [70]. The molecular weight of *Passiflora edulis* peel polysaccharides decreased after Vc-H_2_O_2_ treatment, and their antioxidant capacity was also significantly reduced. It can be concluded that the weakened antioxidant capacity of RGP−2−A decolorized by hydrogen peroxide is related to the decrease in molecular weight.

Furthermore, the relationship between the monosaccharide composition and antioxidant capacity of polysaccharides was mainly related to the galacturonic acid and glucuronic acid of polysaccharides. In this paper, the RGP−2−A galacturonic acid was almost completely eliminated after H_2_O_2_ decolorization. Its antioxidant capacity was also significantly reduced compared to RGP−1−A. This result was also similar to the previous study, where Shao et al. [17] found that galacturonic acid was significantly reduced within the H_2_O_2_-decolorized TCTP compared to the activated carbon decolorized TCTP by comparing *Thesium chinense Turcz* polysaccharides (TCTPs) with different decolorization processes. In the subsequent antioxidant experiments, the antioxidant capacity of TCTP after H_2_O_2_ decolorization was also significantly reduced. Therefore, the content of galacturonic acid is crucial to the antioxidant capacity of polysaccharides.

The specific structure of polysaccharides also affects their biological activity, and in this paper, the sulfate group was also one of the main differences between RGP−1−A and RGP−2−A. Sulfate groups can improve the antioxidant capacity of polysaccharides, and in a study by Marília et al. [71] in which seaweed polysaccharides were modified with sulfation and compared for antioxidant, it was concluded that sulfation modification significantly improved the antioxidant capacity of seaweed polysaccharides.

### 3.12. Protection from H_2_O_2_-Induced Oxidative Damage in IPEC-1 Cells

#### 3.12.1. Cytotoxicity Assay

**Figure 7:**
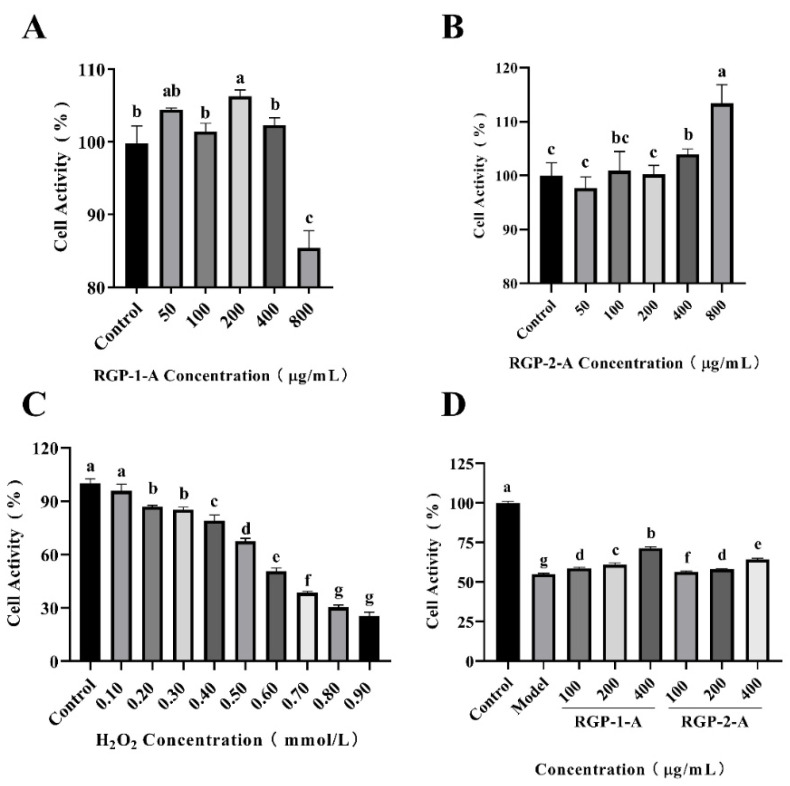
Effects of RGP−1−A (**A**) and RGP−2−A (**B**) on IPEC-1 cell proliferation (versus the untreated control). Cellular oxidative damage model established via the treatment of IPEC-1 cells with different concentrations of H_2_O_2_ (**C**). Effects of RGP−1−A and RGP−2−A on IPEC-1 cell proliferation under oxidative stress (**D**). RGP−1−A: *Rehmannia glutinosa* polysaccharides decolorized by the AB-8 macroporous resin; RGP−2−A: *Rehmannia glutinosa* polysaccharides decolorized by H_2_O_2_. Different lowercase letters on the bar graph indicate significant differences (*p <* 0.05).

The IPEC-1 cell line from jejunum is an experimental model, and this cell line is also widely used in clinical or nutritional re-studies to induce intestinal inflammation or oxidative damage and to assess nutrient function. As seen in Figure 7A, RGP−1−A (50–400 μg/mL) had no cytotoxic effect on IPEC-1 cells, while at 800 μg/mL, there was a significant decrease in cell viability compared to the control (84.96 ± 2.17%, *p <* 0.05) As seen in Figure 7B, RGP−2−A (50–800 μg/mL) had no cytotoxic effect. Therefore, the polysaccharide concentrations were set at 100, 200, and 400 μg/mL for subsequent experiments. The choice of concentration for cell experiments is crucial. RGP−1−A showed significant inhibition of cell activity at 800 μg/mL, probably owing to the presence of glucuronic acid groups within the polysaccharide, and high concentrations of this group have inhibited the growth of various cell lines [26].

#### 3.12.2. Establishment of H_2_O_2_ Damage Model

The intracellular H_2_O_2_ concentration is tightly controlled by various enzymatic and non-enzymatic oxidative systems, and a higher than steady-state intracellular concentration of H_2_O_2_ is thought to induce oxidative stress, which induces growth arrest and cell death [72]. To select the appropriate H_2_O_2_ concentration to model oxidative damage by H_2_O_2_, cells were treated with a concentration gradient of H_2_O_2_ and cell viability was assayed by MTT. Cell viability decreased continuously with increasing H_2_O_2_ concentration (Figure 7C). Compared with the control group, cell viability was 67.40 ± 1.75% and 50.60 ± 1.76% in the 0.5 and 0.6 mmol/L H_2_O_2_-treated groups, respectively (*p <* 0.001). cell viability was also significantly lower in the 0.8 and 0.9 mmol/L H_2_O_2_-treated groups than in the control group (30.328 ± 1.350% and 25.349 ± 2.256%; *p <* 0.001). At these concentrations, the viability of IPEC-1 cells was severely impaired and most cells died. Therefore, we chose a H_2_O_2_ concentration of 0.6 mmol/L for subsequent experiments. Through our previous studies [73], we decided to record the half maximum inhibitory concentration (IC50 value) as the concentration of the test compound that effectively inhibits cell growth by 50% when selecting the optimal H_2_O_2_ concentration. Therefore, we generally choose the concentration of compound with 50–60% cell viability.

#### 3.12.3. Protective Effects of RGP against H_2_O_2_ Injury in IPEC-1 Cells

Cells were first cultured in medium containing the two kinds of RGPs for 12 h. Then, oxidative damage was induced with H_2_O_2_. Finally, cell viability was detected via MTT assays. As shown in Figure 7D, IPEC-1 cell survival in the model group was only 54.85 ± 0.65%. However, this rate significantly increased after the addition of different concentrations of RGP−1−A and RGP−2−A (*p <* 0.001). Hence, RGPs can significantly enhance the survival of IPEC-1 cells against oxidative damage (*p <* 0.001). The cell viability after treatment with 400 μg/mL RGP−1−A and RGP−2−A was 71.43 ± 0.81% and 64.43 ± 0.61%, respectively, indicating that the cell viability was significantly enhanced in the RGP−1−A treatment group (*p <* 0.001).

Based on the above findings, it was postulated that RGP−1−A can more effectively protect IPEC-1 cells from H_2_O_2_-induced damage, consistent with the findings from Jia et al. [57]. This difference could be due to the HRSA of RGP−1−A and the more complex structure of these polysaccharides.

#### 3.12.4. Determination of LDH Activity in Cell Culture Medium

**Figure 8:**
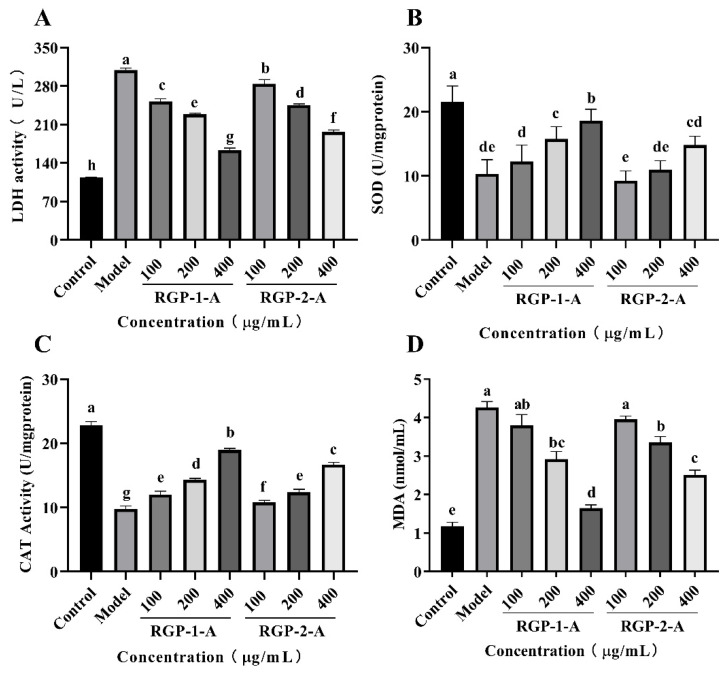
Effects of RGP−1−A and RGP−2−A on H_2_O_2_-induced IPEC-1 cell injury and intracellular antioxidant activity. Levels of LDH (**A**), SOD (**B**), CAT (**C**), and MDA (**D**) were measured using kits. RGP−1−A: *Rehmannia glutinosa* polysaccharides decolorized by the AB-8 macroporous resin; RGP−2−A: *Rehmannia glutinosa* polysaccharides decolorized by H_2_O_2_. Different lowercase letters on the bar graph indicate significant differences (*p <* 0.05).

There are several indicators of cell damage. One key indicator is elevated LDH levels. LDH is a common glycolytic enzyme present in the cytoplasm [74]. Increased LDH release from cells is a sign of oxidative damage and enhanced cell membrane permeability [1], and the amount of leakage could reflect the extent of cell or membrane damage. Therefore, in order to further evaluate the protective effect of RGPs, the LDH level in the culture medium was measured after treatment. Compared with the control group, the 0.6 mmol/L H_2_O_2_ group showed significantly higher LDH activity (*p <* 0.05) (Figure 8A). However, compared with the H_2_O_2_ group, LDH activity was considerably reduced in the 100, 200, and 400 μg/mL RGP groups (*p <* 0.05). RGP−1−A provided a significantly greater reduction in LDH levels than RGP−2−A (*p <* 0.05). Numerous in vitro studies have shown that high levels of H_2_O_2_ can cause severe cellular damage and reduce cell viability [1,75,76]. In line with this, our results showed that H_2_O_2_ (0.6 mM, 6 h) significantly reduced the viability of IPEC-1 cells and increased LDH leakage. Interestingly, pre-incubation of cells with the RGPs 12 h before the addition of H_2_O_2_ significantly inhibited H_2_O_2_-mediated cell death and LDH leakage, demonstrating the anti-apoptotic properties of the RGPs.

#### 3.12.5. Determination of Antioxidant Enzyme Activity

After H_2_O_2_-induced cell injury, the antioxidant enzymes SOD and CAT play crucial roles in restoring the cellular oxidative balance within cells [77,78]. This can efficiently reduce oxidative damage by promoting hydrogen peroxide and lipid peroxide breakdown in human cells [79]. Hence, the effect of RGP on SOD and CAT activity was examined in the present study. SOD and CAT activities were greatly reduced in the H_2_O_2_ model group compared to the control group (*p <* 0.001) (Figure 8B,C), indicating that H_2_O_2_ induced oxidative stress in IPEC-1 cells. SOD and CAT levels in RGP−1−A- and RGP−2−A-treated cells were significantly higher than in the H_2_O_2_ group (*p <* 0.001), and SOD and CAT activities increased the increase in polysaccharide concentration. In a previous study, Huang et al. [80] enhanced the antioxidant protective effect of polysaccharide on cells by sulfating *Mesona chinensis* Benth polysaccharide. Similar to this paper, this property of RGP−1−A may be related to the enhancement of its functional groups and their structural properties (e.g., sulfate groups) on the activity of antioxidant enzymes (CAT and SOD).

#### 3.12.6. Determination of Intracellular Lipid Peroxidation

When the redox balance in the body is disrupted, the main lipid product generated is MDA. Hence, MDA levels can indicate the severity of cell damage [81,82]. As shown in Figure 8D, compared with control group, the MDA level in the H_2_O_2_ group was improved significantly. This difference indicated the presence of significant lipid peroxidation in the H_2_O_2_ group. The MDA level in the RGP−1−A and RGP−2−A treatment groups was significantly lower than the H_2_O_2_ treatment group (*p <* 0.05). Further, the MDA level decreased with an increase in polysaccharide concentration. The MDA level in the RGP−1−A group was significantly lower than that in the RGP−2−A group when the RGP concentration was 400 μg/mL (*p <* 0.05). The difference in MDA between RGP−1−A and RGP−2−A may account for the difference in their chemical composition. Previous studies have shown that sulfation and carboxymethylation modification of polysaccharides can improve their biological activity [76]. In contrast, in this paper, sulfated and carboxymethylated groups in RGP−1−A promoted the production of SOD, and the generated SOD effectively catalyzed the degradation of MDA, thus protecting cells from H_2_O_2_-induced oxidative damage [76,83].

#### 3.12.7. RGP Improves H_2_O_2_-Induced Mitochondrial Function in IPEC-1 Cells

**Figure 9:**
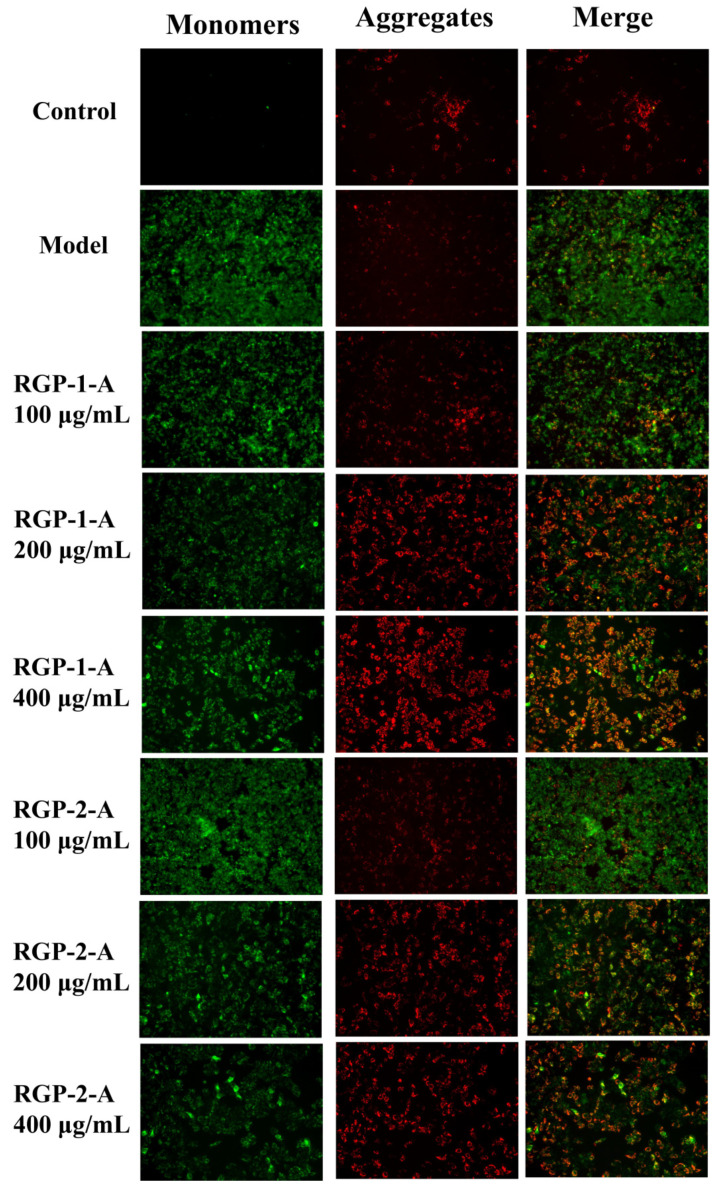
RGPs protected IPEC-1 cells from the H_2_O_2_-induced decrease in mitochondrial membrane potential. IPEC-1 cells were pretreated with 100, 200, or 400 μg/mL of an RGP for 12 h and incubated with 0.8 mmol/L H_2_O_2_ for 8 h. The cells were stained with JC-1, and the morphology of mitochondria was observed using a fluorescence microscope. RGP−1−A: *Rehmannia glutinosa* polysaccharides decolorized by the AB-8 macroporous resin; RGP−2−A: *Rehmannia glutinosa* polysaccharides decolorized by H_2_O_2_.

The reduction in MMP is considered central to the intrinsic apoptotic pathway. This is because H_2_O_2_-induced cell injury leads to ROS production in cells [57], which leads to mitochondrial dysfunction and manifests as changes in MMP [84]. Once the MMP falls, apoptosis becomes inevitable. In this study, the MMP was examined after H_2_O_2_ and polysaccharide treatment using JC-1 staining. The cells in the control group showed red fluorescence after JC-1 staining. However, compared with the control group of IPEC-1 cells, the H_2_O_2_ model group showed significantly lower MMP and more green fluorescence. Compared with the model group, the RGP−1−A and RGP−2−A pretreatment groups showed higher MMP (Figure 9). After pretreatment with RGP−1−A and RGP−2−A for 12 h, the H_2_O_2_-induced decrease in MMP was significantly attenuated in IPEC-1 cells. The recovery effect achieved with RGP−1−A was better than that achieved with RGP−2−A. Similarly, Chen et al. found that *C. paliurus* polysaccharides can attenuate the H_2_O_2_-induced decrease in MMP in L02 cells [85]. Acidic-hydrogenated corn silk polysaccharides have been found to have the same effect [57]. Excessive ROS production can lead to mitochondrial damage, which contributes to various pathologies. Mitochondrial impairment causes defects in lipid homeostasis and bioenergy and ROS production, leading to lipid accumulation and oxidative stress [86]. However, pretreatment with RGPs can attenuate the reduction in MMP by reducing ROS production.

#### 3.12.8. RGP Inhibits Apoptosis and Regulates the Expression of Apoptosis-Related Genes

**Figure 10:**
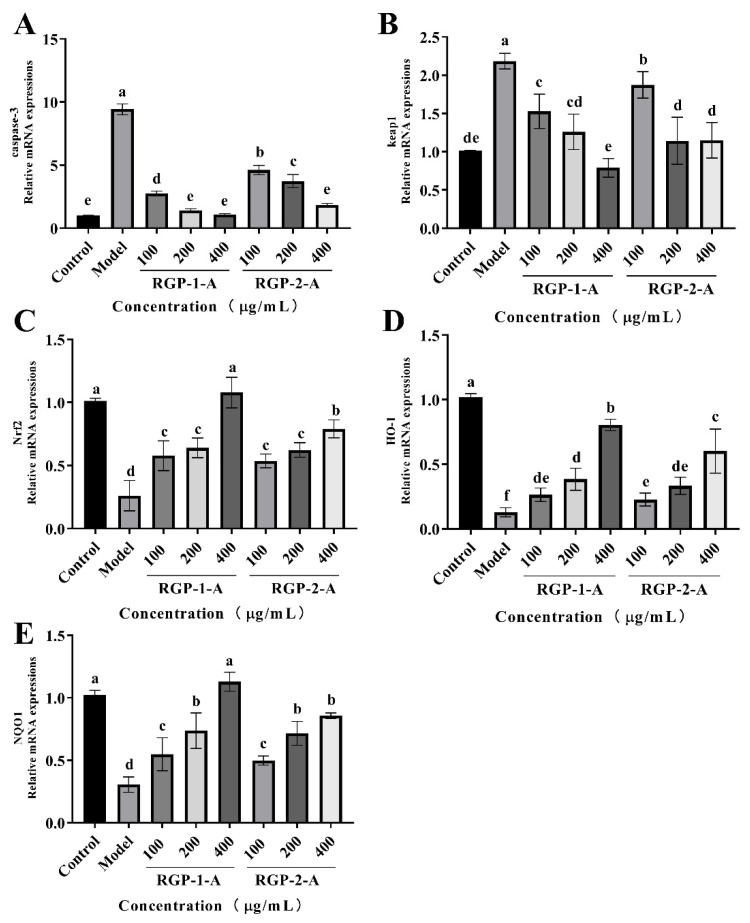
RGP increased the mRNA expression of genes related to the Nrf2/Keap1 signaling pathway in IPEC-1 cells. The mRNA levels of these genes were detected using RT-PCR. (**A**–**E**) Effect of RGP (100, 200, and 400 μg/mL) on the mRNA expression of caspase-3 (**A**), Keap1 (**B**), Nrf2 (**C**), HO-1 (**D**), and NQO1 (**E**). The mRNA levels of all genes were normalized to those of GAPDH. RGP−1−A: *Rehmannia glutinosa* polysaccharides decolorized by the AB-8 macroporous resin; RGP−2−A: *Rehmannia glutinosa* polysaccharides decolorized by H_2_O_2_. Different lowercase letters on the bar graph indicate significant differences (*p <* 0.05).

Caspases regulate apoptosis mainly through the mitochondrial pathway and death receptor pathway, both of which involve caspase-3 [87]. In the death receptor pathway, the DNA cleavage factor is activated via caspase-3, leading to nuclear DNA cleavage and eventually cell death [88,89]. Therefore, by measuring the relative expression of caspase-3 in IPEC-1 cells, it is possible to confirm the protective impact of RGPs against H_2_O_2_-induced oxidative damage. As shown in Figure 10A, H_2_O_2_ treatment promoted apoptosis in IPEC-1 cells, while RGP treatment (100–400 μg/mL) significantly reduced this apoptosis in a dose dependent manner. These results showed that RGP−1−A treatment was more effective in blocking the increase in caspase-3 activity induced by H_2_O_2_, thereby inhibiting subsequent apoptosis signals. In line with this, chestnut shell polysaccharides, which are acidic polysaccharides, have been shown to reduce the H_2_O_2_-induced increase in caspase-3 and apoptosis [29]. Our findings showed that RGP pretreatment can attenuate the H_2_O_2_-induced production of ROS and then block caspase-3 expression and protect cells. Hence, RGPs can prevent apoptosis.

#### 3.12.9. RGP Regulates the Contents of HO-1, NQO1, Keap1, and Nrf2 in H_2_O_2_-Damaged IPEC-1 Cells

Nuclear factor erythroid-2-related factor 2 (Nrf2) can protect cells from oxidative damage and is a key regulator of antioxidant enzymes. Under oxidative stress, Nrf2 is released by the negative regulatory protein Kelch-like erythrocyte-derived protein 1 (Keap1) and transported to the nucleus, where it finally induces the transcription of downstream genes with antioxidant effect, such as HO-1, NQO1, CAT, and SOD. In this study (Figure 10B–E), the H_2_O_2_ group displayed a lower relative expression of Nrf2, HO-1, and NQO1 mRNA than did the control group (*p <* 0.05). However, the relative expression of these mRNAs exhibited a significant dose-dependent increase following RGP treatment (*p <* 0.05). Compared with the control group, the H_2_O_2_ group showed a higher relative expression of Keap1 mRNA (*p <* 0.05). The relative expression of Keap1 mRNA did, however, significantly decline after RGP administration with the decreasing of contents (*p <* 0.05). Overall, RGP−1−A showed the better antioxidant properties between the two polysaccharides. Liu et al. showed that chestnut shell polysaccharides containing a sulfate group can significantly attenuate the reduction in Nrf2 mRNA expression in liver cells caused by H_2_O_2_ injury [29]. Acidic-hydrogenated corn silk polysaccharides have been found to protect IEC-6 cells from H_2_O_2_ damage by increasing the expression of Nrf2 and HO-1 or reducing that of Keap1 [57]. Interestingly, black mulberry (*Morus nigra* L.) polysaccharides can significantly reduce the palmitate-induced decrease in Nrf2, NQO1, and HO-1 mRNA expression, thus mitigating oxidative stress [90]. The Nrf2-Keap1 pathway is an important pathway and can induce the expression of antioxidant enzymes (HO-1, NQO1, SOD, and CAT); remove ROS; and regulate lipid peroxide (MDA) levels [90].

In conclusion, RGPs exert antioxidant effects by promoting the expression of the Nrf2 gene, increasing the expression of downstream genes (HO-1 and NQO1), promoting the production of antioxidant enzymes (SOD and CAT), and reducing intracellular ROS levels. This decrease in intracellular ROS can prevent mitochondrial dysfunction, block the activation of caspase-3, and thereby inhibit apoptosis. Thus, it can be seen that RGP can be used as a beneficial antioxidant active ingredient in food products. There are already *Rehmannia glutinosa* food products, such as porridge, chicken soup, tea, wine, and so on. These foods or health products contain amounts of polysaccharide components, but for medicinal food made directly with the raw *Rehmannia glutinosa* materials, the composition is more complicated and its usage must be controlled. Therefore, the functional factors of *Rehmannia glutinosa* have been finely analyzed to improve their safety.

## 4. Conclusions

In conclusion, compared with polysaccharides decolorized using macroporous resin, those decolorized using H_2_O_2_ had a different Mw, monosaccharide composition, and functional group profile. In this study, TGA showed that the RGP decolorized using H_2_O_2_ had better thermal stability, and XRD analysis showed that both RGPs coexisted in the microcrystalline and amorphous states. Experiments on IPEC-1 cells showed that RGPs can reduce oxidative stress in cells by regulating the Nrf2/Keap1 pathway and reduce the degree of apoptosis by blocking the expression of caspase-3. In vitro and in vivo antioxidant experiments revealed that resin-decolorized RGP had a stronger antioxidant activity. This could be because the molecular structure of the RGP was altered during H_2_O_2_-induced decolorization, which, in turn, altered the biological activity. Although H_2_O_2_ decolorization is more effective, the free radicals generated during H_2_O_2_ decolorization can affect the structure of the RGP. In order to maximize the protection of polysaccharide structure, the conditions for H_2_O_2_ decolorization should be further optimized. In addition, it remains necessary to further explore the mechanism of H_2_O_2_ decolorization of RGPs and to clarify the action site of H_2_O_2_ leading to structural changes of RGPs.

## Figures and Tables

**Table 1:** Molecular weight of different polysaccharides.

Parameters	RGP−1−A	RGP−2−A
Molecular weight, Mw (Da)	18,964	3305
Molecular weight, Mn (Da)	6090	2475
Polydispersity (Mw/Mn)	3.11382	1.33534

**Table 2:** Monosaccharide composition of different polysaccharides.

Monosaccharide Composition (mol %)		
Glucose	45.335	38.006
Galactose	25.873	56.461
Arabinose	10.285	2.470
Galacturonic acid	13.522	1.196
Rhamnose	2.901	0.152
Mannose	0.099	0.227
Ribose	0.036	0.005
Xylose	0.566	0
Fucose	0.521	0.809
Glucuronic acid	0.753	0.675
*N*-acetyl-glucosamine	0.110	0

**Table 3:** Properties of different polysaccharides.

Parameters	RGP−1−A	RGP−2−A
Total sugar (%)	78.24 ± 0.52	81.54 ± 0.79
Sulfate content (%)	11.83 ± 0.80	8.56 ± 1.20
Galacturonic acid (%)	19.02 ± 0.42	1.1 ± 0.27
Zeta-potential (mV)	−15.35 ± 0.45	−8.76 ± 0.19

RGP−1−A: *Rehmannia glutinosa* polysaccharide decolorized by AB-8 macroporous resin; RGP−2−A: *Rehmannia glutinosa* polysaccharide decolorized by H_2_O_2_.

**Table 4:** List of polysaccharide-specific FTIR vibrational modes.

RGP−1−A	RGP−2−A	Functional Group	Reference
3390	3370	hydroxyl group stretching vibration	[18]
2940	2930	hydroxyl group stretching vibrations, C−H stretching and bending vibrations	[36]
1740	NF	stretching vibration of the carbonyl group in carboxylic acid groups (C=O)	[48,49]
1620	1610	asymmetric stretching vibration of C=O	[50]
1430	1410	the vibrations of C−H/CH_2_ group	[51]
1360	1370	asymmetrical S=O stretching vibration	[47,51,52]
1210	1240	the stretching vibration of C−O−C or C−OH	[53]
1140	1140	asymmetrical C−O−S stretching vibration	[22,47]
1090, 1030	1080	pyranose rings	[18,52]
886	875, 834	C−O−SO_3_ group	[22,47,52]
764, 701	796, 692	C−O−C bending vibrations in glycosidic bonds	[49]
534	NF	the characteristic bands of sulfuric acid ester	[54]

NF: Not found in map.

## Data Availability

The data presented in this study are available on request from the corresponding author.

## Supplementary Materials

The following supporting information can be downloaded at: https://www.mdpi.com/article/10.3390/foods11213449/s1, Figure S1: Separation of RGP-1 (**A**) and RGP-2 (**C**) using a DEAE-52 cellulose column. Elution profiles of RGP−1−A (**B**) and RGP−2−A (**D**) separated using a Sephadex G-100 column; Figure S2: X-ray diffraction patterns of RGP−1−A (**A**) and RGP−2−A (**B**); Figure S3: Thermogravimetry analysis patterns of RGP−1−A (**A**) and RGP−2−A (**B**); Table S1: Primer sequences used in quantitative real-time polymerase chain reaction; Table S2: The program for real-time fluorescent quantitative PCR (RT-PCR).

## References

[1] Wen Z., Ma L., Xiang X., Tang Z., Guan R., Qu Y. (2018). Protective effect of low molecular-weight seleno-aminopolysaccharides against H_2_O_2_-induecd oxidative stress in intestinal epithelial cells. Int. J. Biol. Macromol..

[2] Bardaweel S.K., Gul M., Alzweiri M., Ishaqat A., Alsalamat H.A., Bashatwah R.M. (2018). Reactive oxygen species: The dual role in physiological and pathological conditions of the human body. Eurasian J. Med..

[3] Mani S., Swargiary G., Ralph S.J. (2022). Targeting the redox imbalance in mitochondria: A novel mode for cancer therapy. Mitochondrion.

[4] Feng J., Cai Z., Zhang X., Chen Y., Chang X., Wang X., Qin C., Yan X., Ma X., Zhang J. (2020). The effects of oral *Rehmannia glutinosa* polysaccharide administration on immune responses, antioxidant activity and resistance against *Aeromonas hydrophila* in the common carp, *Cyprinus carpio* L. Front. Immunol..

[5] Liu C., Ma R., Wang L., Zhu R., Liu H., Guo Y., Zhao B., Zhao S., Tang J., Li Y. (2017). Rehmanniae radix in osteoporosis: A review of traditional chinese medicinal uses, phytochemistry, pharmacokinetics and pharmacology. J. Ethnopharmacol..

[6] Jensen S.R., Li H., Albach D.C., Gotfredsen C.H. (2008). Phytochemistry and molecular systematics of triaenophora rupestris and *Oreosolen wattii* (scrophulariaceae). Phytochemistry.

[7] Zhou J., Xu G., Yan J., Li K., Bai Z., Cheng W., Huang K. (2015). *Rehmannia glutinosa* (gaertn.) Dc. *Polysaccharide ameliorates* hyperglycemia, hyperlipemia and vascular inflammation in streptozotocin-induced diabetic mice. J. Ethnopharmacol..

[8] Kwak M., Yu K., Lee P.C., Jin J. (2018). *Rehmannia glutinosa* polysaccharide functions as a mucosal adjuvant to induce dendritic cell activation in mediastinal lymph node. Int. J. Biol. Macromol..

[9] Wu C., Shan J., Feng J., Wang J., Qin C., Nie G., Ding C. (2019). Effects of dietary radix rehmanniae preparata polysaccharides on the growth performance, immune response and disease resistance of *Luciobarbus capito*. Fish Shellfish Immun..

[10] Chen G., Chen R., Chen D., Ye H., Hu B., Zeng X., Liu Z. (2019). Tea polysaccharides as potential therapeutic options for metabolic diseases. J. Agr. Food Chem..

[11] Mulinari Turin De Oliveira N., Barbosa Da Luz B., Schneider V.S., Barbosa Da Costa Filho H., Sérgio De Araujo Sousa P., Fernanda De Paula Werner M., Henrique Loiola Ponte De Souza M., Almeida Rocha J., Antonio Duarte Nicolau L., Mach Côrtes Cordeiro L. (2022). Dietary polysaccharides from guavira pomace, a co-product from the fruit pulp industry, display therapeutic application in gut disorders. Food Res. Int..

[12] Bell T.J., Draper S.L., Centanni M., Carnachan S.M., Tannock G.W., Sims I.M. (2018). Characterization of polysaccharides from feijoa fruits (*Acca sellowiana* berg.) And their utilization as growth substrates by gut commensal bacteroides species. J. Agr. Food Chem..

[13] Masuda Y., Matsumoto A., Toida T., Oikawa T., Ito K., Nanba H. (2009). Characterization and antitumor effect of a novel polysaccharide from *Grifola frondosa*. J. Agr. Food Chem..

[14] Rupérez P., Ahrazem O., Leal J.A. (2002). Potential antioxidant capacity of sulfated polysaccharides from the edible marine brown seaweed *Fucus vesiculosus*. J. Agr. Food Chem..

[15] Pan F., Su T., Liu Y., Hou K., Chen C., Wu W. (2018). Extraction, purification and antioxidation of a polysaccharide from *Fritillaria unibracteata* var. Wabuensis. Int. J. Biol. Macromol..

[16] Shao L., Sun Y., Liang J., Li M., Li X. (2020). Decolorization affects the structural characteristics and antioxidant activity of polysaccharides from thesium chinense turcz: Comparison of activated carbon and hydrogen peroxide decolorization. Int. J. Biol. Macromol..

[17] Xie J., Shen M., Nie S., Li C., Xie M. (2011). Decolorization of polysaccharides solution from *Cyclocarya paliurus* (batal.) Iljinskaja using ultrasound/H_2_O_2_ process. Carbohyd. Polym..

[18] Hu Z., Zhou H., Li Y., Wu M., Yu M., Sun X. (2019). Optimized purification process of polysaccharides from *Carex meyeriana* kunth by macroporous resin, its characterization and immunomodulatory activity. Int. J. Biol. Macromol..

[19] Zhang Y., Campbell R., Drake M., Zhong Q. (2015). Decolorization of cheddar cheese whey by activated carbon. J. Dairy Sci..

[20] Belwal T., Li L., Yanqun X., Cravotto G., Luo Z. (2020). Ultrasonic-assisted modifications of macroporous resin to improve anthocyanin purification from a *Pyrus communis* var. Starkrimson extract. Ultrason. Sonochem..

[21] Gong Y., Ma Y., Cheung P.C., You L., Liao L., Pedisić S., Kulikouskaya V. (2021). Structural characteristics and anti-inflammatory activity of uv/H_2_O_2_-treated algal sulfated polysaccharide from *Gracilaria lemaneiformis*. Food Chem. Toxicol..

[22] Sharma K., Kumar M., Waghmare R., Suhag R., Gupta O.P., Lorenzo J.M., Prakash S., Radha, Rais N., Sampathrajan V. (2022). Moringa (*Moringa oleifera* Lam.) Polysaccharides: Extraction, characterization, bioactivities, and industrial application. Int. J. Biol. Macromol..

[23] Huang G., Chen F., Yang W., Huang H. (2021). Preparation, deproteinization and comparison of bioactive polysaccharides. Trends Food Sci. Tech..

[24] Liu J., Li Y., Pu Q., Qiu H., Di D., Cao Y. (2022). A polysaccharide from *Lycium barbarum* L.: Structure and protective effects against oxidative stress and high-glucose-induced apoptosis in arpe-19 cells. Int. J. Biol. Macromol..

[25] Nataraj A., Govindan S., Ramani P., Subbaiah K.A., Sathianarayanan S., Venkidasamy B., Thiruvengadam M., Rebezov M., Shariati M.A., Lorenzo J.M. (2022). Antioxidant, anti-tumour, and anticoagulant activities of polysaccharide from *Calocybe indica* (apk2). Antioxidants.

[26] Khan B.M., Qiu H., Wang X., Liu Z., Zhang J., Guo Y., Chen W., Liu Y., Cheong K. (2019). Physicochemical characterization of *Gracilaria chouae* sulfated polysaccharides and their antioxidant potential. Int. J. Biol. Macromol..

[27] Yang Y., Qiu Z., Li L., Vidyarthi S.K., Zheng Z., Zhang R. (2021). Structural characterization and antioxidant activities of one neutral polysaccharide and three acid polysaccharides from *Ziziphus jujuba* cv. Hamidazao: A comparison. Carbohyd. Polym..

[28] Liu H., Fang Y., Li Y., Ma L., Wang Q., Xiao G., Zou C. (2021). Characterization of pcs-2a, a polysaccharide derived from chestnut shell, and its protective effects against H_2_O_2_-induced liver injury in hybrid grouper. Int. J. Biol. Macromol..

[29] Jridi M., Mezhoudi M., Abdelhedi O., Boughriba S., Elfalleh W., Souissi N., Nasri R., Nasri M. (2018). Bioactive potential and structural characterization of sulfated polysaccharides from bullet tuna (*Auxis rochei*) by-products. Carbohyd. Polym..

[30] Mohammed J.K., Mahdi A.A., Ahmed M.I., Ma M., Wang H. (2020). Preparation, deproteinization, characterization, and antioxidant activity of polysaccharide from *Medemia argun* fruit. Int. J. Biol. Macromol..

[31] Qiao H., Shao H., Zheng X., Liu J., Liu J., Huang J., Zhang C., Liu Z., Wang J., Guan W. (2021). Modification of sweet potato (*Ipomoea batatas* lam.) Residues soluble dietary fiber following twin-screw extrusion. Food Chem..

[32] López-Legarda X., Rostro-Alanis M., Parra-Saldivar R., Villa-Pulgarín J.A., Segura-Sánchez F. (2021). Submerged cultivation, characterization and in vitro antitumor activity of polysaccharides from *Schizophyllum radiatum*. Int. J. Biol. Macromol..

[33] Rincón E., Espinosa E., García-Domínguez M.T., Balu A.M., Vilaplana F., Serrano L., Jiménez-Quero A. (2021). Bioactive pectic polysaccharides from bay tree pruning waste: Sequential subcritical water extraction and application in active food packaging. Carbohyd. Polym..

[34] Yi J., Li X., Wang S., Wu T., Liu P. (2022). Steam explosion pretreatment of *Achyranthis bidentatae* radix: Modified polysaccharide and its antioxidant activities. Food Chem..

[35] Ballesteros L.F., Cerqueira M.A., Teixeira J.A., Mussatto S.I. (2015). Characterization of polysaccharides extracted from spent coffee grounds by alkali pretreatment. Carbohyd. Polym..

[36] Cao J., Yang J., Wang Z., Lu M., Yue K. (2020). Modified citrus pectins by uv/H_2_O_2_ oxidation at acidic and basic conditions: Structures and in vitro anti-inflammatory, anti-proliferative activities. Carbohyd. Polym..

[37] Li J., Kusche-Gullberg M. (2016). Heparan sulfate: Biosynthesis, structure, and function. Int. Rev. Cel. Mol. Bio..

[38] Yao W., Liu M., Chen X., You L., Ma Y., Hileuskaya K. (2022). Effects of uv/H_2_O_2_ degradation and step gradient ethanol precipitation on *Sargassum fusiforme* polysaccharides: Physicochemical characterization and protective effects against intestinal epithelial injury. Food Res. Int..

[39] Chen X., You L., Ma Y., Zhao Z., Kulikouskaya V. (2021). Influence of uv/H_2_O_2_ treatment on polysaccharides from *Sargassum fusiforme*: Physicochemical properties and raw 264.7 cells responses. Food Chem. Toxicol..

[40] Chen S., Liu H., Yang X., Li L., Qi B., Hu X., Ma H., Li C., Pan C. (2020). Degradation of sulphated polysaccharides from *Grateloupia livida* and antioxidant activity of the degraded components. Int. J. Biol. Macromol..

[41] Chen B., Shi M., Cui S., Hao S., Hider R.C., Zhou T. (2016). Improved antioxidant and anti-tyrosinase activity of polysaccharide from *Sargassum fusiforme* by degradation. Int. J. Biol. Macromol..

[42] Mcconaughy S.D., Stroud P.A., Boudreaux B., Hester R.D., Mccormick C.L. (2008). Structural characterization and solution properties of a galacturonate polysaccharide derived from *Aloe vera* capable of in situ gelation. Biomacromolecules.

[43] Koocheki A., Hesarinejad M.A., Mozafari M.R. (2022). *Lepidium perfoliatum* seed gum: Investigation of monosaccharide composition, antioxidant activity and rheological behavior in presence of salts. Chem. Biol. Technol. Agric..

[44] Sun Y., Gong G., Guo Y., Wang Z., Song S., Zhu B., Zhao L., Jiang J. (2018). Purification, structural features and immunostimulatory activity of novel polysaccharides from *Caulerpa lentillifera*. Int. J. Biol. Macromol..

[45] Hu W., Chen S., Wu D., Zheng J., Ye X. (2019). Ultrasonic-assisted citrus pectin modification in the bicarbonate-activated hydrogen peroxide system: Chemical and microstructural analysis. Ultrason. Sonochem..

[46] Souza B.W.S., Cerqueira M.A., Bourbon A.I., Pinheiro A.C., Martins J.T., Teixeira J.A., Coimbra M.A., Vicente A.A. (2012). Chemical characterization and antioxidant activity of sulfated polysaccharide from the red seaweed *Gracilaria birdiae*. Food Hydrocolloid..

[47] Chokboribal J., Tachaboonyakiat W., Sangvanich P., Ruangpornvisuti V., Jettanacheawchankit S., Thunyakitpisal P. (2015). Deacetylation affects the physical properties and bioactivity of acemannan, an extracted polysaccharide from *Aloe vera*. Carbohyd. Polym..

[48] Fernando I.P.S., Sanjeewa K.K.A., Samarakoon K.W., Lee W.W., Kim H., Kim E., Gunasekara U.K.D.S., Abeytunga D.T.U., Nanayakkara C., de Silva E.D. (2017). Ftir characterization and antioxidant activity of water soluble crude polysaccharides of sri lankan marine algae. ALGAE.

[49] López-Legarda X., Arboleda-Echavarría C., Parra-Saldívar R., Rostro-Alanis M., Alzate J.F., Villa-Pulgarín J.A., Segura-Sánchez F. (2020). Biotechnological production, characterization and in vitro antitumor activity of polysaccharides from a native strain of *Lentinus crinitus*. Int. J. Biol. Macromol..

[50] Chaouch M.A., Hammi K.M., Dhahri M., Mansour M.B., Maaroufi M.R., Le Cerf D., Majdoub H. (2018). Access to new anticoagulant by sulfation of pectin-like polysaccharides isolated from opuntia *Ficus indica cladodes*. Int. J. Biol. Macromol..

[51] Prado-Fernández J., Rodríguez-Vázquez J.A., Tojo E., Andrade J.M. (2003). Quantitation of κ-, ι- and λ-carrageenans by mid-infrared spectroscopy and pls regression. Anal. Chim. Acta.

[52] Xu C., Leppänen A., Eklund P., Holmlund P., Sjöholm R., Sundberg K., Willför S. (2010). Acetylation and characterization of spruce (*Picea abies*) galactoglucomannans. Carbohyd. Res..

[53] Harris M.J., Turvey J.R. (1970). Sulphates of monosaccharides and derivatives: Part viii. Infrared spectra and optical rotations of some glycoside sulphates. Carbohyd. Res..

[54] Figueroa F.A., Abdala-Díaz R.T., Pérez C., Casas-Arrojo V., Nesic A., Tapia C., Durán C., Valdes O., Parra C., Bravo-Arrepol G. (2022). Sulfated polysaccharide extracted from the green algae *Codium bernabei*: Physicochemical characterization and antioxidant, anticoagulant and antitumor activity. Mar. Drugs.

[55] Singh B., Sharma V. (2016). Designing galacturonic acid/arabinogalactan crosslinked poly(vinyl pyrrolidone)- co-poly(2-acrylamido-2-methylpropane sulfonic acid) polymers: Synthesis, characterization and drug delivery application. Polymer.

[56] Jia Y., Wang Y., Li R., Li S., Zhang M., He C., Chen H. (2021). The structural characteristic of acidic-hydrolyzed corn silk polysaccharides and its protection on the H_2_O_2_-injured intestinal epithelial cells. Food Chem..

[57] Chen H., Zeng J., Wang B., Cheng Z., Xu J., Gao W., Chen K. (2021). Structural characterization and antioxidant activities of bletilla striata polysaccharide extracted by different methods. Carbohyd. Polym..

[58] Su Y., Li L. (2020). Structural characterization and antioxidant activity of polysaccharide from four auriculariales. Carbohyd. Polym..

[59] Guo Q., Cui S.W., Wang Q., Hu X., Kang J., Yada R.Y. (2012). Structural characterization of a low-molecular-weight heteropolysaccharide (glucomannan) isolated from *Artemisia sphaerocephala* krasch. Carbohyd. Res..

[60] Ding S., Yan Z., Liu H., Chen P., Shi S., Chang M. (2022). Structural characterization and antitumor activity of a polysaccharide extracted from perilla frutescens var. Frutescens. Ind. Crop. Prod..

[61] Chi A., Li H., Kang C., Guo H., Wang Y., Guo F., Tang L. (2015). Anti-fatigue activity of a novel polysaccharide conjugates from ziyang green tea. Int. J. Biol. Macromol..

[62] Chen Y., Jiang X., Xie H., Li X., Shi L. (2018). Structural characterization and antitumor activity of a polysaccharide from *Ramulus mori*. Carbohyd. Polym..

[63] Liu X., Gu L., Zhang G., Liu H., Zhang Y., Zhang K. (2022). Structural characterization and antioxidant activity of polysaccharides extracted from chinese yam by a cellulase-assisted method. Process Biochem..

[64] Huo J., Wu J., Huang M., Zhao M., Sun W., Sun X., Zheng F. (2020). Structural characterization and immuno-stimulating activities of a novel polysaccharide from huangshui, a byproduct of chinese baijiu. Food Res. Int..

[65] Li Y., Lin D., Jiao B., Xu C., Qin J., Ye G., Su G. (2015). Purification, antioxidant and hepatoprotective activities of polysaccharide from *Cissus pteroclada hayata*. Int. J. Biol. Macromol..

[66] Buathongjan C., Israkarn K., Sangwan W., Outrequin T., Gamonpilas C., Methacanon P. (2020). Studies on chemical composition, rheological and antioxidant properties of pectin isolated from riang (*Parkia timoriana* (dc.) Merr.) Pod. Int. J. Biol. Macromol..

[67] Ge Q., Mao J., Guo X., Zhou Y., Gong J., Mao S. (2013). Composition and antioxidant activities of four polysaccharides extracted from *Herba lophatheri*. Int. J. Biol. Macromol..

[68] Fimbres-Olivarria D., Carvajal-Millan E., Lopez-Elias J.A., Martinez-Robinson K.G., Miranda-Baeza A., Martinez-Cordova L.R., Enriquez-Ocaa F., Valdez-Holguin J.E. (2018). Chemical characterization and antioxidant activity of sulfated polysaccharides from *Navicula* sp. Food Hydrocolloid..

[69] Li X., Zhang G., Li J., Jiang T., Chen H., Li P., Guan Y. (2021). Degradation by vc—H_2_O_2_,characterization and antioxidant activity of polysaccharides *Frompassiflora edulis* peel. J. Food Process. Pres..

[70] Fernandesnegreiros M.M., Batista L.A.N.C., Silva V.R.L., Araujo S.D., Paiva A.A.O., Paiva W.S., Machado R.I.A., Sousa J.F.L.D., de Lima P.D., Vitoriano J.D.O. (2020). Gallic acid-laminarin conjugate is a better antioxidant than sulfated or carboxylated laminarin. Antioxidants.

[71] Gülden M., Jess A., Kammann J., Maser E., Seibert H. (2010). Cytotoxic potency of H_2_O_2_ in cell cultures: Impact of cell concentration and exposure time. Free Radic. Biol. Med..

[72] Khan S.U., Ullah F., Mehmood S., Fahad S., Ahmad Rahi A., Althobaiti F., Dessoky E.S., Saud S., Danish S., Datta R. (2021). Antimicrobial, antioxidant and cytotoxic properties of *Chenopodium glaucum* L. PLoS ONE.

[73] Van Wilpe S., Koornstra R., Den Brok M., De Groot J.W., Blank C., De Vries J., Gerritsen W., Mehra N. (2020). Lactate dehydrogenase: A marker of diminished antitumor immunity. Oncoimmunology.

[74] Seto S.W., Chang D., Ko W.M., Zhou X., Kiat H., Bensoussan A., Lee S.M.Y., Hoi M.P.M., Steiner G.Z., Liu J. (2017). Sailuotong prevents hydrogen peroxide (H_2_O_2_)-induced injury in ea.hy926 cells. Int. J. Mol. Sci..

[75] Xie L., Huang Z., Qin L., Yu Q., Chen Y., Zhu H., Xie J. (2022). Effects of sulfation and carboxymethylation on cyclocarya paliurus polysaccharides: Physicochemical properties, antitumor activities and protection against cellular oxidative stress. Int. J. Biol. Macromol..

[76] Hu Y., Lu S., Li Y., Wang H., Shi Y., Zhang L., Tu Z. (2022). Protective effect of antioxidant peptides from grass carp scale gelatin on the H_2_O_2_-mediated oxidative injured hepg2 cells. Food Chem..

[77] Yan F., Chen L., Chen W., Zhao L., Lu Q., Liu R. (2021). Protective effect of procyanidin a-type dimers against H_2_O_2_-induced oxidative stress in prostate du145 cells through the mapks signaling pathway. Life Sci..

[78] Zhuang C., Xu N., Gao G., Ni S., Miao K., Li C., Wang L., Xie H. (2016). Polysaccharide from *Angelica sinensis* protects chondrocytes from H_2_O_2_-induced apoptosis through its antioxidant effects in vitro. Int. J. Biol. Macromol..

[79] Huang L., Huang M., Shen M., Wen P., Wu T., Hong Y., Yu Q., Chen Y., Xie J. (2019). Sulfated modification enhanced the antioxidant activity of mesona chinensis benth polysaccharide and its protective effect on cellular oxidative stress. Int. J. Biol. Macromol..

[80] Moniruzzaman M., Ghosal I., Das D., Chakraborty S.B. (2018). Melatonin ameliorates H_2_O_2_-induced oxidative stress through modulation of erk/akt/nfkb pathway. Biol. Res..

[81] Garcia Y.J., Rodríguez-Malaver A.J., Peñaloza N. (2005). Lipid peroxidation measurement by thiobarbituric acid assay in rat cerebellar slices. J. Neurosci. Meth..

[82] Ren G., Li K., Hu Y., Yu M., Qu J., Xu X. (2015). Optimization of selenizing conditions for seleno-lentinan and its characteristics. Int. J. Biol. Macromol..

[83] Chen P., Liu H., Ji H., Sun N., Feng Y. (2019). A cold-water soluble polysaccharide isolated from *Grifola frondosa* induces the apoptosis of hepg2 cells through mitochondrial passway. Int. J. Biol. Macromol..

[84] Chen X., Wang X., Shen M., Chen Y., Yu Q., Yang J., Xie J. (2022). Combined rna-seq and molecular biology technology revealed the protective effect of cyclocarya paliurus polysaccharide on H_2_O_2_-induced oxidative damage in l02 cells thought regulating mitochondrial function, oxidative stress and pi3k/akt and mapk signaling pathways. Food Res. Int..

[85] Sharma V., Pathak K. (2019). Liquisolid system of paclitaxel using modified polysaccharides: In vitro cytotoxicity, apoptosis study, cell cycle analysis, in vitro mitochondrial membrane potential assessment, and pharmacokinetics. Int. J. Biol. Macromol..

[86] Walsh J.G., Cullen S.P., Sheridan C., Lüthi A.U., Gerner C., Martin S.J. (2008). Executioner caspase-3 and caspase-7 are functionally distinct proteases. Proc. Natl. Acad. Sci. USA.

[87] Suresh K., Carino K., Johnston L., Servinsky L., Machamer C.E., Kolb T.M., Lam H., Dudek S.M., An S.S., Rane M.J. (2019). A nonapoptotic endothelial barrier-protective role for caspase-3. Am. J. Physiol.. Lung Cell. Mol. Physiol..

[88] Galluzzi L., Vitale I., Abrams J.M., Alnemri E.S., Baehrecke E.H., Blagosklonny M.V., Dawson T.M., Dawson V.L., El-Deiry W.S., Fulda S. (2012). Molecular definitions of cell death subroutines: Recommendations of the nomenclature committee on cell death 2012. Cell Death Differ..

[89] Chen W., Lu Y., Hu D., Mo J., Ni J. (2021). Black mulberry (*Morus nigra* L.) Polysaccharide ameliorates palmitate-induced lipotoxicity in hepatocytes by activating nrf2 signaling pathway. Int. J. Biol. Macromol..

[90] Liu H., Fang Y., Zou C. (2021). Pomelo polysaccharide extract inhibits oxidative stress, inflammation, and mitochondrial apoptosis of *Epinephelus coioides*. Aquaculture.

