# Supplementary material for: Structural Characteristics of Rehmannia glutinosa Polysaccharides Treated Using Different Decolorization Processes and Their Antioxidant Effects in Intestinal Epithelial Cells
Source: Foods. 2022 Oct 31;11(21):3449. doi: 10.3390/foods11213449 (PMC9657679; doi:10.3390/foods11213449)
Supplement: Supplementary file 1 [file foods-11-03449-s001.zip › foods-1904627-supplementary.pdf]

Supplementary data

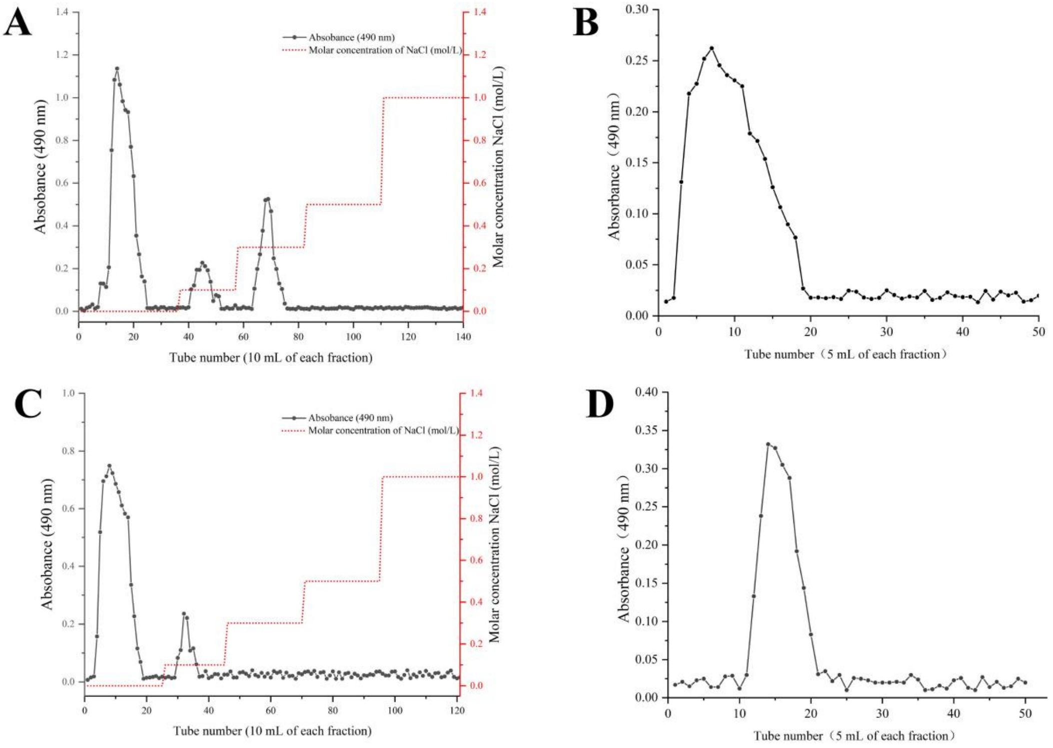

Figure S1. Separation of RGP-1 (A) and RGP-2 (C) using a DEAE-52 cellulose column. Elution profiles of RGP−1−A (B) and RGP−2−A (D) separated using a Sephadex G-100 column. RGP−1−A: *Rehmannia glutinosa* polysaccharide decolorized by AB-8 macroporous resin, RGP−2−A: *Rehmannia glutinosa* polysaccharide decolorized by H_2O_2.

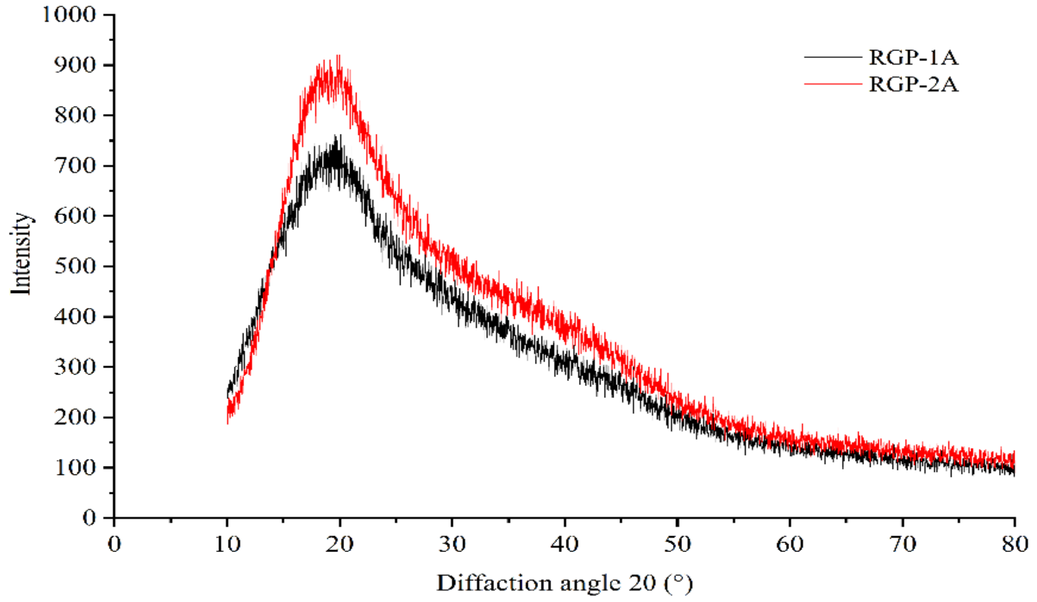

Figure S2. X-ray diffraction patterns of RGP−1−A (A) and RGP−2−A (B).

RGP−1−A: *Rehmannia glutinosa* polysaccharide decolorized by AB-8 macroporous resin, RGP−2−A: *Rehmannia glutinosa* polysaccharide decolorized by H_2O_2.

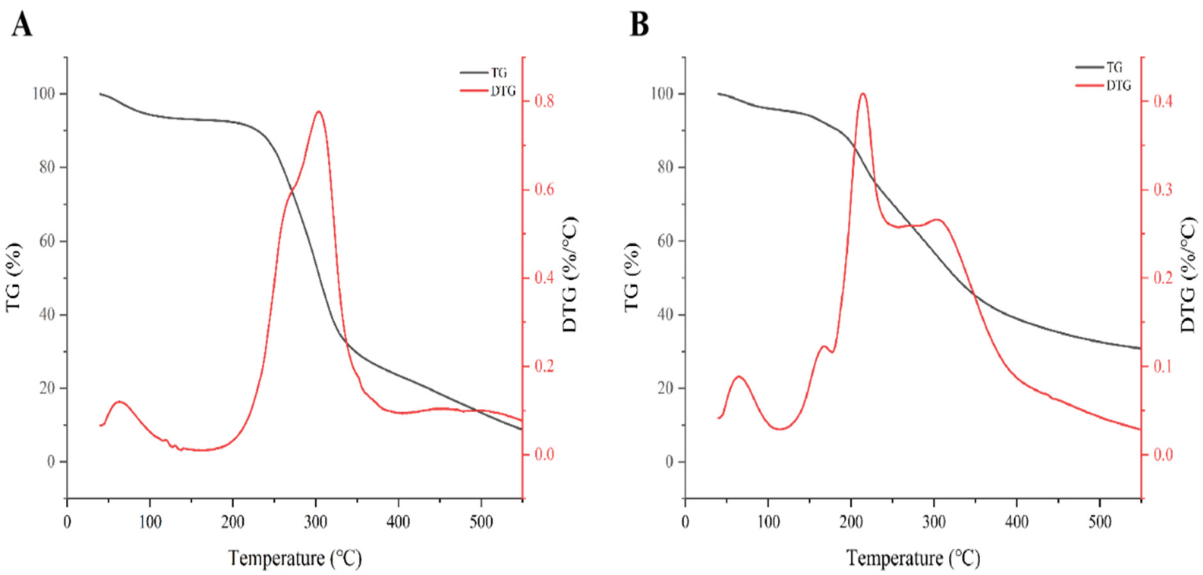

Figure S3. Thermogravimetry analysis patterns of RGP−1−A (A) and RGP−2−A (B). RGP−1−A: *Rehmannia glutinos* polysaccharide decolorized by AB-8 macroporous resin, RGP−2−A: *Rehmannia glutinosa* polysaccharide decolorized by H_2O_2.

Table S1 Primer sequences used in quantitative real-time polymerase chain reaction.

Gene		Primer sequence 5′-3′	PCR product size (bp)
Keap1	Forward primer	ATGGCGGGGCCTCTGA	114
	Reverse primer	CTCAGGGGCAGAAATTGGGT	
Nrf2	Forward primer	AGCGGATTGCTCGTAGACAG	155
	Reverse primer	TCAATCAAATCCATGTCCTTGGC	
caspase-3	Forward primer	GCCAGGAATAGTAACCAGGTGCTG	112
	Reverse primer	TGTGGGATTGAGACGGACAGTGG	
HO-1	Forward primer	CTGAGAATGCCGAGTTCAT	156
	Reverse primer	GGAAGTAGAGGGGCGTGTAG	
NQO1	Forward primer	TGGTGGAGTCGGACCTCTATG	287
	Reverse primer	CATGGCAGCGTAAGTGTAAGC	
GAPDH	Forward primer	GCCATCACAGCCACACAGAAGA	206
	Reverse primer	CGGCAGGTCAGGTCAACAACAG	

Table S2. The program for real-time fluorescent quantitative PCR (RT-PCR)

**Reaction stage**	**Specific response**	**Cycle**	**temperature**	**time**
1	Predegeneration	1	95℃	30 s
2	Cyclic reaction	40	95℃	10 s
			60℃	30 s
3	Melting curve	1	95℃	15 s
			60℃	60 s
			95℃	15 s
